# A comprehensive atlas of Aggrecan, Versican, Neurocan and Phosphacan expression across time in wildtype retina and in retinal degeneration

**DOI:** 10.1038/s41598-022-11204-w

**Published:** 2022-05-04

**Authors:** A. Matsuyama, A. A. Kalargyrou, A. J. Smith, R. R. Ali, R. A. Pearson

**Affiliations:** 1 Ocular Cell and Gene therapy Group, Centre for Gene Therapy and Regenerative Medicine, King’s College London, 8th Floor, Tower Wing, Guy’s Hospital, London, SE1 9RT UK; 2 University College London Institute of Ophthalmology, 11-43 Bath Street, London, EC1V 9EL UK; 3 Present Address: RIKEN Center for Integrative Medical Sciences, 1-7-22 Suehiro-cho, Tsurumi-ku, Yokohama City, Kanagawa 230-0045 Japan

**Keywords:** Cell death in the nervous system, Retina, Macular degeneration

## Abstract

As photoreceptor cells die during retinal degeneration, the surrounding microenvironment undergoes significant changes that are increasingly recognized to play a prominent role in determining the efficacy of therapeutic interventions. Chondroitin Sulphate Proteoglycans (CSPGs) are a major component of the extracellular matrix that have been shown to inhibit neuronal regrowth and regeneration in the brain and spinal cord, but comparatively little is known about their expression in retinal degeneration. Here we provide a comprehensive atlas of the expression patterns of four individual CSPGs in three models of inherited retinal degeneration and *wildtype* mice. In *wildtype* mice, Aggrecan presented a biphasic expression, while Neurocan and Phosphacan expression declined dramatically with time and Versican expression remained broadly constant. In degeneration, Aggrecan expression increased markedly in *Aipl1*^*-/-*^ and *Pde6b*^*rd1/rd1*^, while Versican showed regional increases in the periphery of *Rho*^*-/-*^ mice. Conversely, Neurocan and Phosphacan broadly decrease with time in all models. Our data reveal significant heterogeneity in the expression of individual CSPGs. Moreover, there are striking differences in the expression patterns of specific CSPGs in the diseased retina, compared with those reported following injury elsewhere in the CNS. Better understanding of the distinct distributions of individual CSPGs will contribute to creating more permissive microenvironments for neuro-regeneration and repair.

## Introduction

Chondroitin sulphate proteoglycans (CSPGs) are major components of the extracellular matrix (ECM) in the central nervous system (CNS) and can exert effects on cell adhesion, motility, axonal outgrowth, synaptic plasticity and neural regeneration^1–3^. They are typically upregulated in response to injury and in a wide range of neurodegenerative diseases. Importantly, they have been shown to reduce the efficacy of various therapeutic approaches^4–6^. CSPGs consist of a core protein covalently attached to long glycosaminoglycan (GAG) carbohydrate chains^7^. There are 16 different types of CSPGs, of which Neurocan, Brevican, Phosphacan, Aggrecan and Versican are most common within the nervous system^7^. CSPGs exert much of their inhibitory function via their GAG chain composition^3^, however the core proteins themselves also inhibit neurite outgrowth^8^, an effect that persists after the removal of GAGs^9^. Understanding the specific changes in expression of individual CSPGs in response to injury or disease is crucial for creating extracellular environments that are permissive for regenerative therapies^4^.

Aggrecan is one of the major CSPGs expressed in the nervous system and is a constituent of perineuronal nets (PNNs)^3,10,11^. It has been studied extensively in spinal cord injury where it prevents Schwan cell migration following transplantation by inhibiting integrin-mediated cell adhesion^12^, as well as inhibiting endogenous axonal growth in injured spinal cord in vivo^13^. Neurocan is similarly elevated around acute lesions in the CNS and active multiple sclerosis plaque edges^14–17^ as well as in many experimental models of acute injury in the brain and is commonly associated with inhibition of neurite outgrowth^14,18,19^.

Little is known about CSPG expression in the retina, although there are indications of unique roles of individual CSPGs: In the rodent retina, Phosphacan protein levels increase from late embryonic to late postnatal stages but are markedly reduced in the adult retina^20^. Conversely, Aggrecan increases with age^21,22^. In the normal rat retina, Neurocan, Aggrecan and Versican are differentially distributed at embryonic, postnatal and adult stages^21^. With respect to the human eye, a recent study using human embryonic stem cell-derived retinal organoids indicated that BREVICAN and VERSICAN were found throughout the retina across much of development, although a downregulation of *VCAN,* and a concomitant upregulation of *BCAN* expression was observed in late developmental stages^23^. In the human adult eye, VERSICAN and AGGRECAN are found throughout the neural retina, choroid and sclera^22^.

Much less is known about CSPG expression in the diseased retina. Using the pan-CSPG marker CS-56, we have previously shown that CSPG deposition varies markedly both across degeneration and between different murine models of photoreceptor degeneration^24^. However, the changes in expression of specific CSPGs following different types ocular injury, and particularly inherited disease, are poorly characterised. Aggrecan was shown to be upregulated in rat models of retinal dystrophy^25^. Conversely, *Aggrecan* expression decreased, while *Brevican* and *Phosphacan* mRNA levels were unchanged in the neural retina following induced injury (ischaemia) in the rat retina, but all three were significantly increased in the optic nerve^26^.

To explore the potential heterogeneity in the microenvironment between different degenerative disease models, we examined four individual CSPGs, Neurocan, Aggrecan, Phosphacan and Versican. We present a comprehensive assessment of protein and mRNA expression both across time and with degeneration in three murine models of retinal degeneration of differing severity. Our findings revealed a marked heterogeneity in the expression of different CSPG core proteins and this can arise even between models with similar rates of photoreceptor loss. This data provides a valuable resource and reference for future studies but also shows the vital importance of understanding the specific microenvironment changes associated with a given disease type when assessing the suitability and timings of therapeutic interventions.

## Methods

### Animals

Mouse lines used in the study include *wildtype C57BL/6 J* (Harlan, UK), *Rho*^*-/-*^ (on a *C57BL/6 J* background; kind gift from P. Humphries, Trinity College Dublin, Republic of Ireland), *Aipl1*^*-/-*^ (kind gift from T. Li, National Eye Institute, USA; formerly Harvard Medical School at the Massachusetts Eye and Ear Infirmary, USA), and *Pde6b*^*rd1/rd1*^ (line originally on *C3H/HeJ* background [Harlan, UK], but backcrossed with *C57BL/6 J* to remove confounding *Gpr179* (Bipolar cell) mutant alleles; see Nishiguchi et al*.* 2015^27^. All experiments have been conducted in accordance with the Policies on the Use of Animals and Humans in Neuroscience Research, revised and approved by the ARVO Statement for Use of Animals in the Ophthalmic Research, under the regulation of the UK Home Office Animals (Scientific Procedures) Act 1986. Mice were used according to the NC3R ARRIVE guidelines. Mice were maintained in the animal facility at University College London or King’s College London in individually ventilated cages and given access to nesting material and food and water ad libitum. They were kept on a standard 12/12 h light/ dark cycle and at the same light levels throughout the study and used at the ages specified in the text. Animals of both sexes were used in this study without discrimination.

### Time points and degeneration models

We used the *Aipl1*^*-/-*^ model of Lebers congenital amourosis, and the *Pde6b*^*rd1/rd1*^ and *Rho*^*-/-*^ models of Retinitis Pigmentosa, all of which have mutations in genes expressed by photoreceptors. We examined each model at ‘early’ (defined as ONL > 70% of the thickness of the wild-type retinae), ‘mid’ (ONL thickness of 30–70% of wildtype), and ‘advanced’ stage degeneration (ONL < 30%), as previously defined^1,24^ and summarised in Table 1. For *Aipl1*^*-/-*^ mice, which exhibit a very rapid degeneration that starts almost as soon as the retina is formed, we used P10 for early (no degeneration, 10 rows of nuclei in photoreceptor layer), P14 for mid (photoreceptors layer is reduced by half) and P19-P21 for advanced (a single layer of photoreceptor nuclei remains) stages, in accordance with previously published data^28,29^. *Pde6b*^*rd1/rd1*^ is a model of fairly fast degeneration and was examined at P10 weeks for early (no degeneration, 10 rows of nuclei in photoreceptor layer), 3 weeks for mid (photoreceptors layer is reduced by half) and 6 weeks for advanced (a single layer of photoreceptor nuclei remains) stages. *Rho*^*-/-*^ degenerates more slowly and was examined at early (3–4 weeks, 9 rows of nuclei in photoreceptor layer), 6 weeks for mid (photoreceptors layer is reduced by half) and 10–12 wks for advanced (2–3 rows of nuclei in the photoreceptor layer) stages. *C57BL/6 J wildtype* mice were used as background- and age-matched controls. We used 6 time points for wildtype mice to match the different time points used for the degenerative models: Postnatal day (P)10, when neurogenesis is largely completed but the synaptic layers are forming; P14, when synaptogenesis is nearly complete; 3 weeks; 6 weeks, 3 months and 6 months. Of note, in order to compare the expression levels in the postnatal period, real-time qPCR was performed for P10 *Rho*^*-/-*^ mice in addition to the later degeneration stages.

**Table 1:** Summary of timepoints used in retinal degeneration models.

Model	Degeneration (progression rate)	Early (timepoint)	Mid (timepoint)	Advanced (timepoint)
*Aipl1*^*-/-*^	Very fast	P10	P14	3 weeks
*Pde6b*^*rd1/rd1*^	Moderate	P10	3 weeks	6 weeks
*Rho*^*-/-*^	Moderate to slow	3–4 weeks	6 weeks	10–12 weeks

### Immunohistochemistry and histology

**Figure 1:**
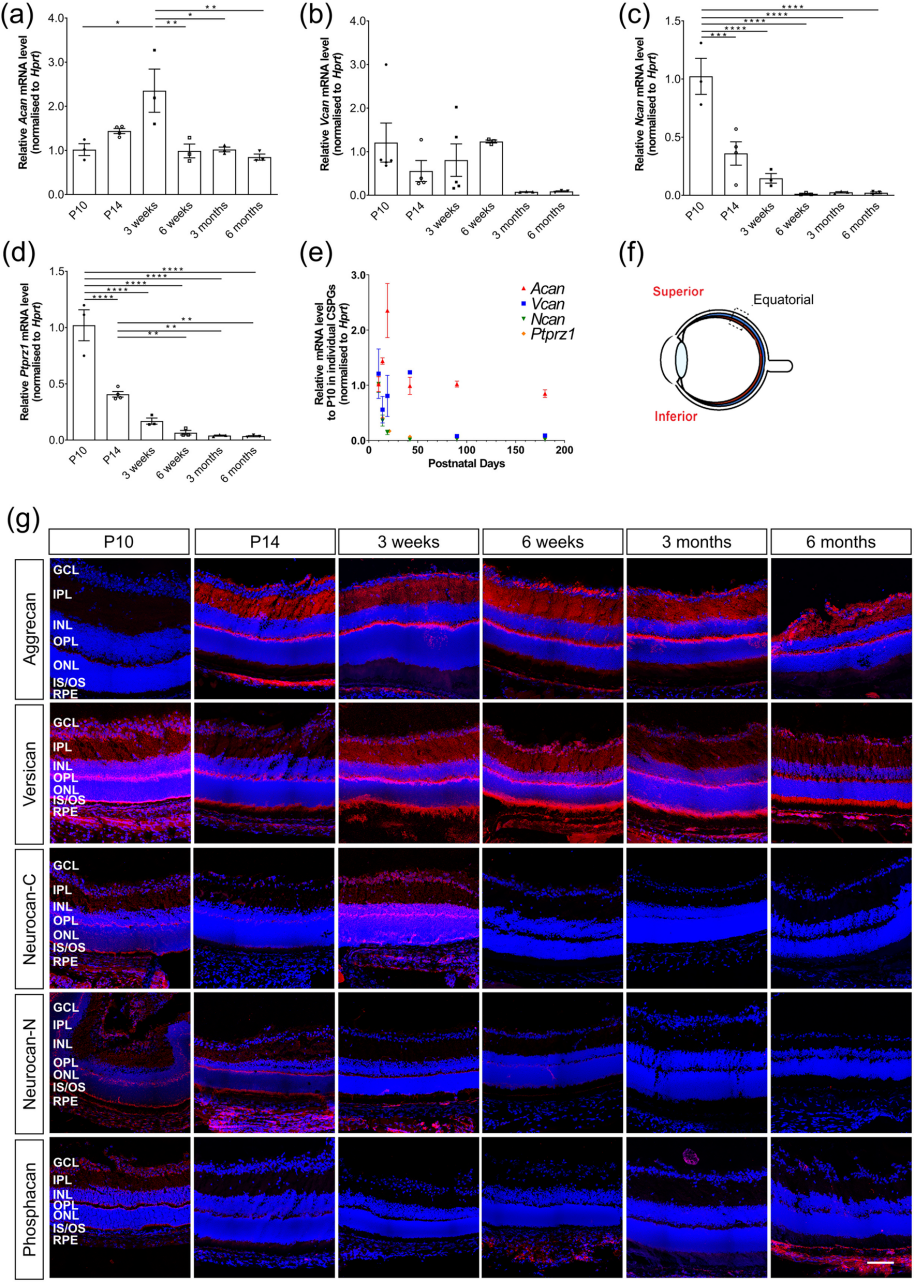
Expression patterns of Aggrecan, Versican, Neurocan and Phosphacan in *wildtype* mice over time. (**a**)–(**d**) histograms showing mean (+ /− SEM) mRNA levels of *Acan* (**a**), *Vcan* (**b**), *Ncan* (**c**) and *Ptprz1* (**d**) in whole retinae. (**e**) best-fit curves summarizing the changes in expression of all four CSPGs over time. (Lognormal for *Acan* and *Vcan*, one phase decay for *Ncan* and *Ptprz1*.) (**f**) a schematic of the regions where immunostaining images were taken. (**g**) Immunostaining for Aggrecan, Versican, Neurocan and Phosphacan *(red)* over time. (**a**) Relative expression of *Acan* mRNA increased between P10 and 3 weeks, decreasing thereafter and remained at a similar level throughout adulthood. (**b**) *Vcan* expression was fairly constant across the time points examined, reducing at 3 and 6 months, although inter-sample variation was high. (**c**) (**d**) *Ncan* and *Ptprz1* mRNA expression decreased significantly with time. (**e**) Neurocan-C and -N fractions and Versican were sparsely distributed throughout all the layers of retina when they are expressed. Phosphacan and Aggrecan were restricted mostly to the GCL, IPL and OPL. Images show confocal maximum projection images (MIPs) of the superior retina in the equatorial region. Scale bar, 100 µm. *p < 0.05, **p < 0.01, ***p < 0.001, ****p < 0.0001 (one-way ANOVA test with Bonferroni’s correction). ONL—outer nuclear layer; OPL—outer plexiform layer; INL—inner nuclear layer; IPL—inner plexiform layer; GCL—ganglion cell layer. Nuclei are counter stained with Dapi -*blue*; CSPGs -*red*.

Animals were euthanized and a small burn was administered to the sclera overlying the orbit to provide a landmark for the superior retina. Eyes were enucleated and eye cups prepared in phosphate-buffered saline (PBS) and then carefully orientated and embedded in a standardized fashion in OCT (TissueTek) without fixation. Embedded eyecups were frozen and left at − 20 °C at least overnight before being cut as transverse sections, 18 μm thick. In order to avoid oblique cuts, all images shown are from the central most region of the eye, immediately adjacent to the optic nerve (Fig. 1f). Specific details for each antibody and protocols used for immunohistochemistry can be found in Supplementary Table S1. Briefly, cryosections were air-dried for 15–30 min and washed in PBS. Sections were pre-blocked for 1–1.5 h at room temperature (RT) in a blocking solution before being incubated with appropriate primary antibody overnight (o/n) at 4 °C and at RT for 1–1.5 h. After rinsing with PBS, sections were incubated with secondary antibody (1:400) for 2–2.5 h at RT, rinsed and counter-stained with DAPI (0.5 μg/ml). Negative controls omitted the primary antibody. Qualitative assessments made using immunohistochemistry are based on N > 3 independent eyes per time point, per model, respectively.

### Chondroitinase ABC treatment

The lyophilized ChABC enzyme (Sigma, C2905) was reconstituted in 0.01% BSA in dH_2_O solution to a concentration [1U/ml] and stored at − 80 °C. Prior staining the enzyme was further diluted in an activation buffer TrisHCL [100 mM], (pH = 8) & Sodium Acetate [120 mM] as per Table S1, in a final volume of 100 μl/slide. The slides were covered with parafilm, to avoid evaporation and then incubated in a humidity chamber in a HybEZ™ II Oven (ACD Bio) at 37 °C for 3 h.

### Confocal microscopy

**Figure 2:**
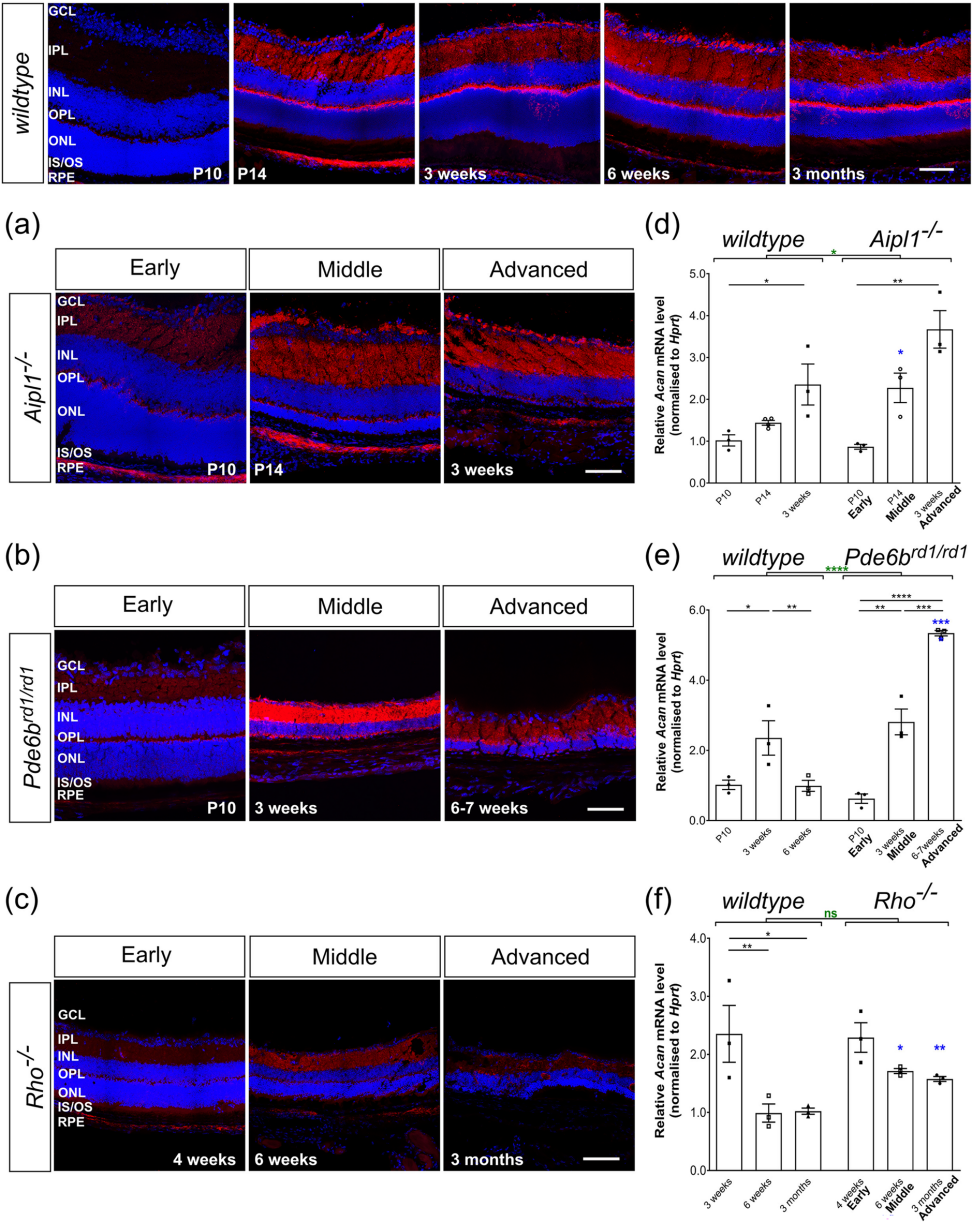
Aggrecan expression is increased in the *Aipl1*^*-/-*^ and *Pde6b*^*rd1/rd1*^ but not *Rho*^*-/-*^ models of retinal degeneration. (**a**)–(**c**), Immunostaining for Aggrecan (*red*) was mainly seen in the OPL, IPL, and GCL with relatively little staining in ONL in all models. Increasing expression was most notable in the IPL, compared to early time-points, in *wildtype*, *Aipl1*^*-/-*^ and *Pde6b*^*rd1/rd1*^ retinae. (**d**)–(**f**), *Acan* mRNA levels were dramatically elevated with disease progression, showing increased expression in (**d**) P14 (mid-stage) *Aipl1*^*-/-*^ and (**e**) 6 week (advanced-stage) *Pde6b*^*rd1/rd1*^ retinae compared to age-matched *wildtype* mice. (**f**) *Acan* mRNA levels decreased in adulthood in *Rho*^*-/-*^ retinae but remained higher than age-matched *wildtype* retinae. N.B. mRNA levels shown for *wildtype* are the same as in Fig. 1b. Scale bar, 100 µm. *p < 0.05, **p < 0.01, ***p < 0.001, ****p < 0.0001 (one-way ANOVA test with Bonferroni’s correction, *black*; unpaired t-test for age-matched comparisons between wildtype and disease model, *blue*; two-way ANOVA test applied for assessments of change over time, *red*). ONL—outer nuclear layer; OPL—outer plexiform layer; INL—inner nuclear layer; IPL—inner plexiform layer; GCL—ganglion cell layer. Nuclei are counter stained with Dapi (*blue*).

**Figure 3:**
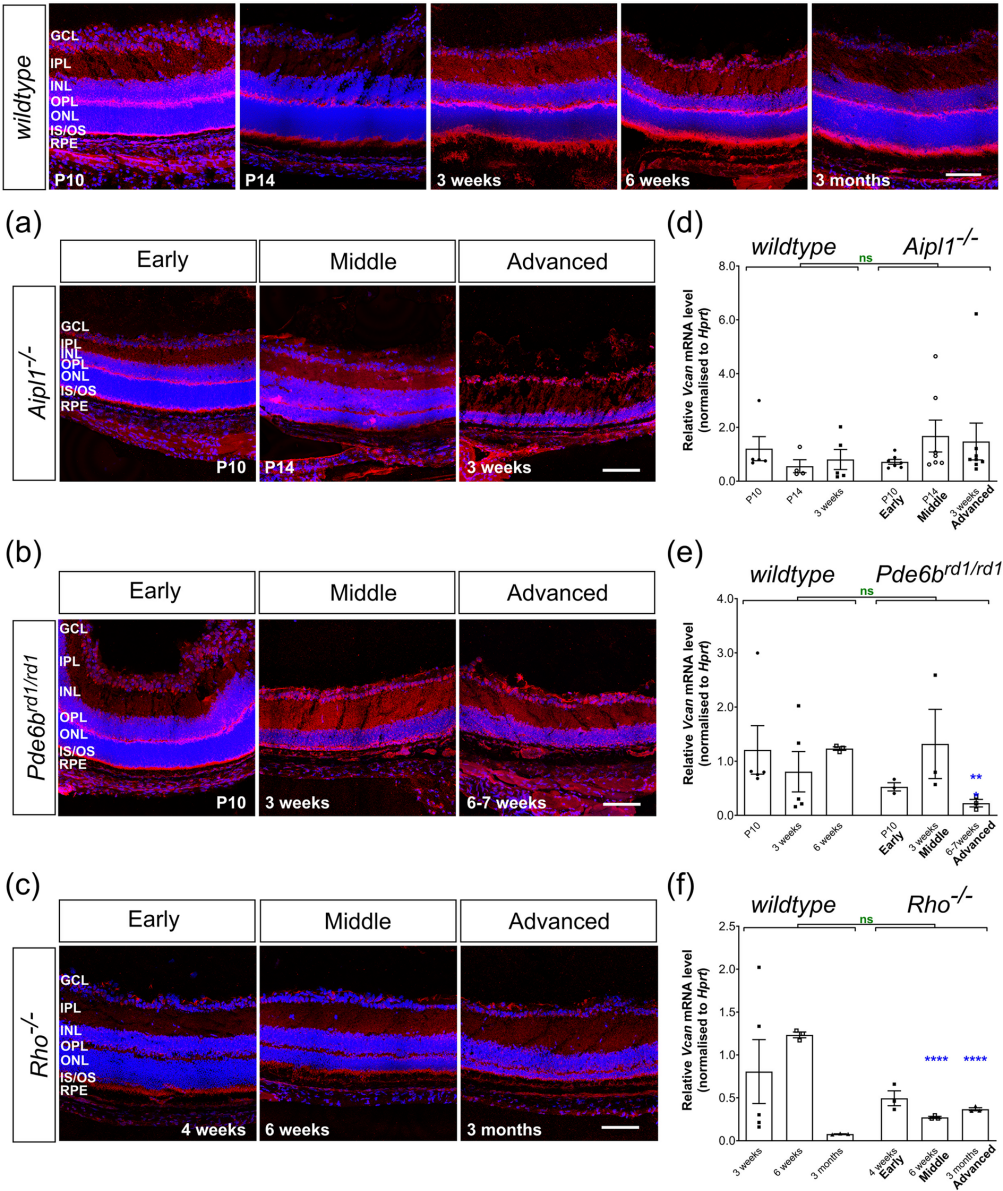
Versican expression remains similar to *wildtype* during degeneration. (**a**)–(**c**), Immunolabelling for Versican (*red*) was detected in all the layers of the retina in *wildtype*, *Aipl1*^*-/-*^ and *Pde6b*^*rd1/rd1*^ with relatively strong signals in the INL and IPL, particularly in mid and advanced stage *Pde6b*^*rd1/rd1*^ and advanced-stage *Rho*^*-/-*^ retinae. (**d**)–(**f**) *Vcan* mRNA levels were largely unchanged in *Aipl1*^*-/-*^ and *Pde6b*^*rd1/rd1*^ retinae (with the exception of a reduction at 6 weeks age of *Pde6b*^*rd1/rd1*^ mice). (**f**) In *Rho*^*-/-*^, *Vcan* mRNA expression remained similar between P10 and 3 months of age, decreasing slightly, but remained significantly higher than age-matched *wildtype* mice. N.B. mRNA levels shown for *wildtype* are the same as in Fig. 1c. Scale bar, 100 µm. *p < 0.05, **p < 0.01, ***p < 0.001, ****p < 0.0001 (one-way ANOVA test with Bonferroni’s correction, *black*; unpaired t-test for age-matched comparisons between *wildtype* and disease model, *blue*; two-way ANOVA test applied for assessments of change over time, *red*). ONL—outer nuclear layer; OPL—outer plexiform layer; INL—inner nuclear layer; IPL—inner plexiform layer; GCL—ganglion cell layer. Nuclei are counter stained with Dapi (*blue*).

**Figure 4:**
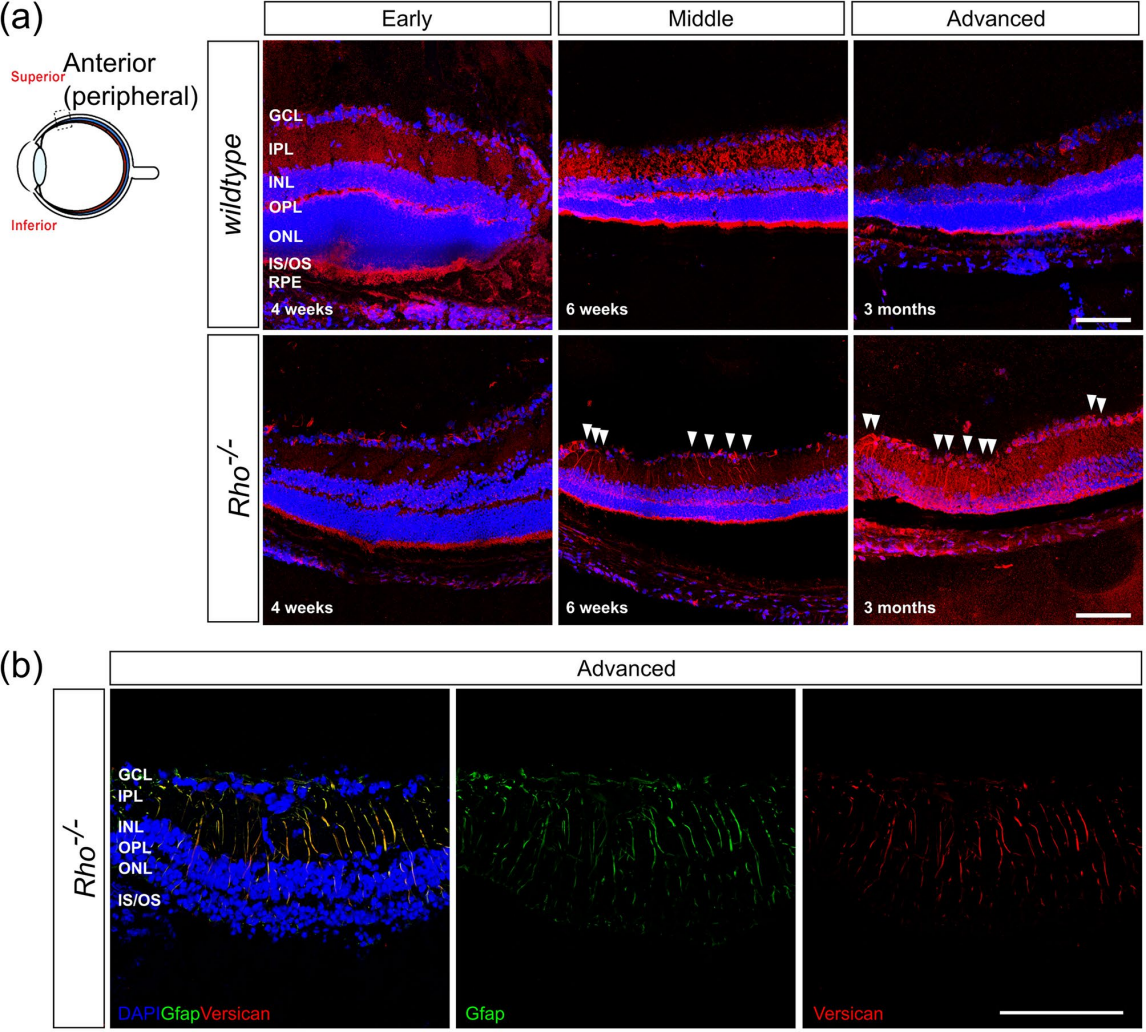
Versican colocalizes with Müller glia in the peripheral retina in advanced stage degeneration of *Rho*^*-/-*^ mice. (a) Versican + radial processes (*red*) were observed in mid- to advanced-stage degeneration of *Rho*^-/-^ mice in the peripheral retina (white arrows) but not in age-matched *wildtype* mice, or in any other model examined. (b) Versican + processes (*red*) co-labelled for the reactive glial marker, Glial Fibrillary Acidic Protein (GFAP; *green*). Image was acquired at × 63 magnification and a single optical section is shown. Scale bar, 100 µm. Nuclei are counter stained with Dapi (*blue*).

**Figure 5:**
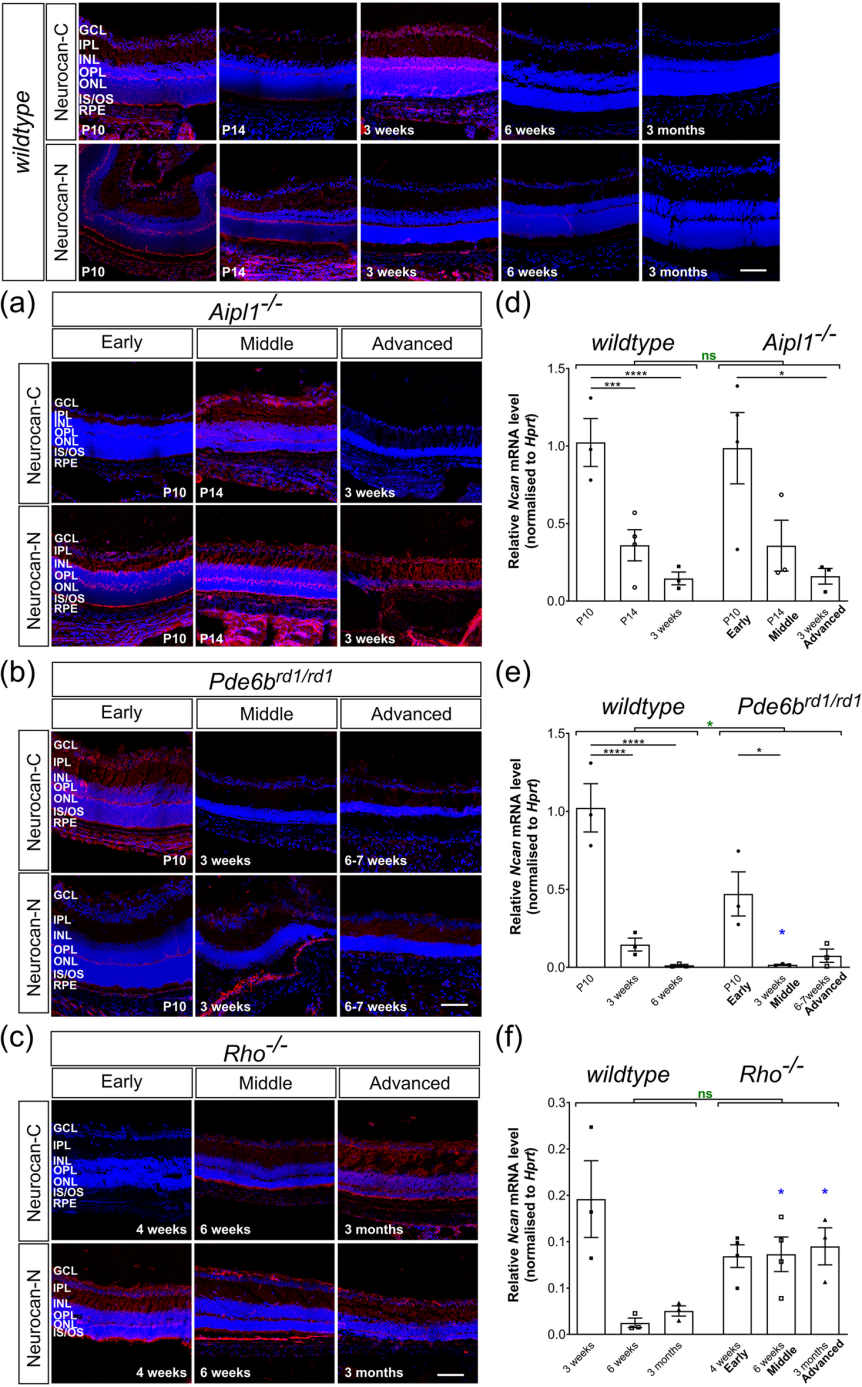
Neurocan expression decreases with time and is unaffected by degeneration. Neurocan immunolabelling (*red*) was distributed throughout all the layers of the retina and low levels of mRNA transcripts were observed in adult retinae in all models. mRNA levels shown for *wildtype* are the same as in Fig. 1d. (**a**) (**b**) Immunolabelling of the Neurocan C-terminal fraction typically decreased in intensity in *Aipl1*^*-/-*^ and *Pde6b*^*rd1/rd1*^ mice with time, while that of the Neurocan N-terminal fraction remained constant in these models. (**d**) (**e**) mRNA levels of *Ncan* decreased with time in *Aipl1*^*-/-*^, *Pde6b*^*rd1/rd1*^ and *wildtype* mice. (**f**) *Ncan* mRNA and (**c**) immunolabelling for both Neurocan-C and -N were largely unchanged across degeneration in *Rho*^*-/-*^ mice. Scale bar, 100 µm. *p < 0.05, **p < 0.01, ***p < 0.001, ****p < 0.0001 (one-way ANOVA test with Bonferroni’s correction, *black*; unpaired t-test for age-matched comparisons between wildtype and disease model, *blue*; two-way ANOVA test applied for assessments of change over time, *red*). ONL—outer nuclear layer; OPL—outer plexiform layer; INL—inner nuclear layer; IPL—inner plexiform layer; GCL—ganglion cell layer. Nuclei are counter stained with Dapi (*blue*).

**Figure 6:**
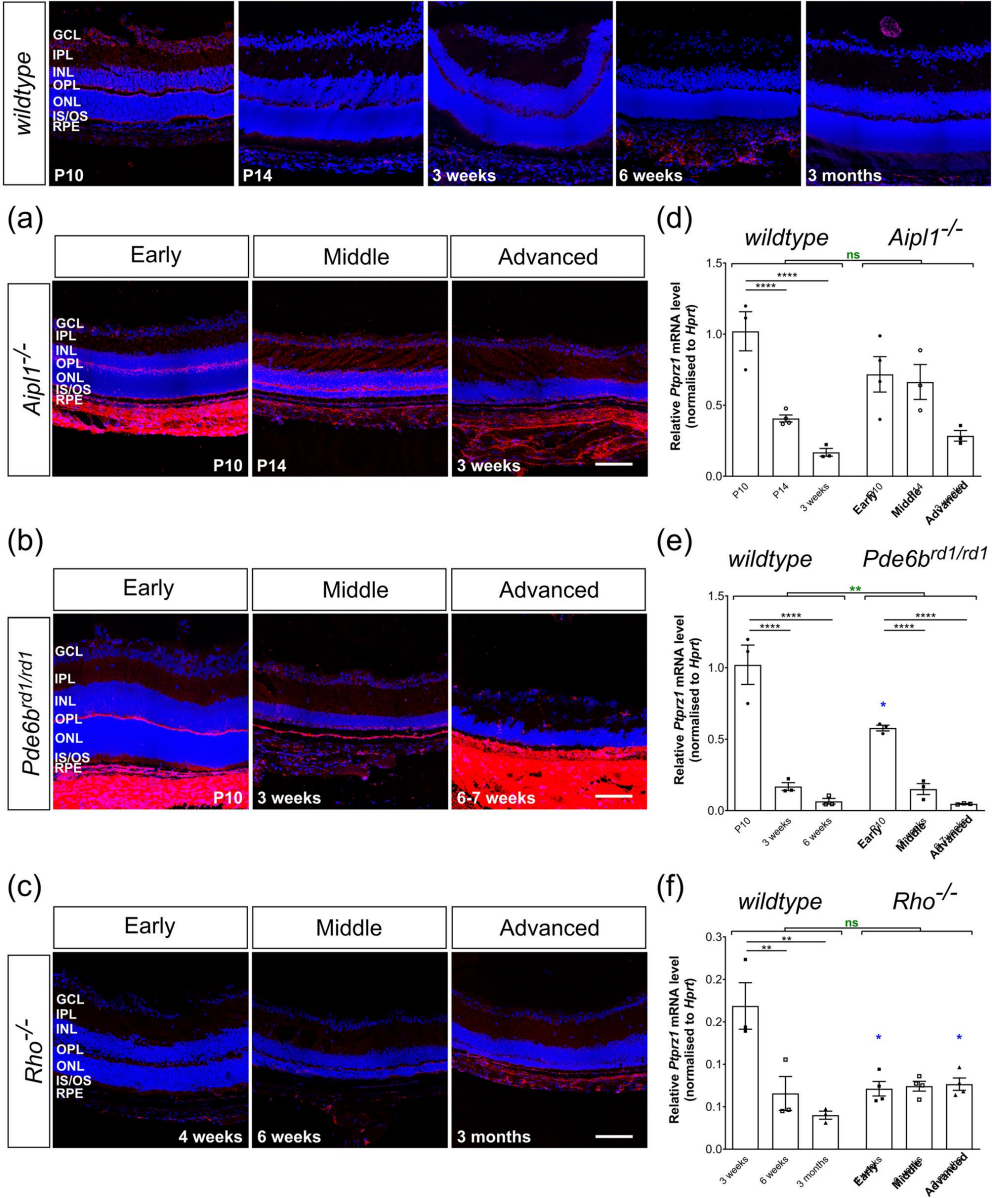
Phosphacan expression decreases with time and is unaffected by degeneration. (**a**)–(**c**) Immunolabelling for Phosphacan (*red*) was mostly restricted to GCL, IPL and OPL. In P10 wildtype, labelling was particularly evident at the outer margin of the OPL and the edge of the ONL itself. In (**a**) *Aipl1*^*-/-*^ and (**b**) *Pde6b*^*rd1/rd1*^ mice, labelling was most prominent in the OPL and RPE, compared to age-matched *wildtype*. (**c**) In *Rho*^*-/-*^ retinae, labelling was weak at all time-points. (**d**)–(**f**) *Ptprz1* mRNA expression was significantly decreased with time in all models including *wildtype* retinae. N.B. mRNA levels shown for *wildtype* are the same as in Fig. 1e. Scale bar, 100 µm. *p < 0.05, **p < 0.01, ***p < 0.001, ****p < 0.0001 (one-way ANOVA test with Bonferroni’s correction, *black*; parametric t-test for age-matched comparisons between *wildtype* and disease model, *blue*; two-way ANOVA test applied for assessments of change over time, *red*). ONL—outer nuclear layer; OPL—outer plexiform layer; INL—inner nuclear layer; IPL—inner plexiform layer; GCL—ganglion cell layer. Nuclei are counter stained with Dapi (*blue*).

Retinal sections were viewed on a confocal microscope (Leica TCS SPE, Leica Microsystems, Milton Keynes, UK). Unless otherwise stated, images show merged maximum intensity projection (MIP) images of xyz confocal stacks through retinal sections. Images are montages acquired using the tile scan function except co-staining. Individual xy images were acquired using a 2-frame average (1 frame for DAPI) at 1 μm intervals, and all taken at × 40 magnification, unless otherwise stated. Images were taken in both the superior (presented in Main Figs. 2, 3, 4, 5, 6) and inferior (presented in Supplementary Figs. S1–S4, S6, S7) retina at standardized regions in the equatorial region, and the superior retina in anterior margin (denoted as ‘peripheral’ in the images) immediately adjacent to the optic nerve (see schematics in figures). Images were acquired within 7 days of completing immunostaining and the same laser intensity, gain and offset settings were used across all sections for any given marker. Note that in order to allow direct visual comparison between wildtype data sets and those from the different degenerating models in different figures, images from wildtype retinae are duplicated in a number of figures. Schematics denoting regions imaged are adapted from our previous publication^24^.

### RNA extraction and reverse transcription

Retinal tissues were carefully dissected, snap frozen in liquid nitrogen and kept in − 80 °C until extracted. Total RNA was isolated using DNA/RNA mini kit (Qiagen), following manufacturer’s instructions. Total RNA (700 µg) was reverse transcribed to obtain cDNA using QuantiTect Reverse Transcription kit, following manufacturer’s instructions. Briefly, total RNA was incubated for 15 min at 42 °C with gDNA Wipeout Buffer to eliminate genomic DNA, and the RNA samples was reverse transcribed using Quantiscript Reverse Transcriptase, Quantiscript RT Buffer, and RT Primer Mix at 42 °C for 1 h. The reaction was inactivated at 95 °C for 3 min.

### Real-time qPCR

TaqMan analysis was used to measure mRNA levels of the target genes. To measure mRNA levels of Neurocan (*Ncan*) and Phosphacan (*Ptprz1*), the following primers and probes (TaqMan Universal ProbeLibrary; Roche) were used at final concentration of 200 nM and 100 nM, respectively: *Hprt*; Fw TCCTCCTCAGACCGCTTTT, Rev CCTGGTTCATCATCGCTAATC, Probe number 95. *Ncan*; Fw GCACAGAGCCAATGCTACC, Rev GCCCGATAATGGAACACG, Probe number 3. *Ptprz1*; Fw GGCACTCAGGAGTATCCAACA, Rev GACCAATACGAGACTCATGGCTA, Probe number 34. For Aggrecan (*Acan*) and Versican (*Vcan*), TaqMan Gene Expression Assays (Thermo Fisher Scientific) were used with the following probes (1 µl per reaction): Hprt; Mm01318741_m1, *Acan*; Mm01317794_m1, *Vcan*; Mm01283060_g1. The probes, primers and cDNA were mixed with PerfeCTa® qPCR FastMix II® with ROX (VWR International Ltd) at the total volume of 20 µl per reaction. Amplification of the selected genes from each sample was performed with in three parallel runs on a 96-well reaction plate. The reactions were performed using QuantStudio 6 Flex System (Thermo Fisher Scientific) with the following protocol: 2 min at 50 °C, 10 min polymerase activation at 95 °C and 40 cycles of 15 s denaturation at 95 °C, 1 min annealing and extension at 60 °C.

### Statistics

Relative mRNA expression of the target gene at each time point was calculated using the comparative 2^ΔΔCT^ method (Livak and Schmittgen, 2001), normalised to *Hprt* and relative to the levels at P10 in *wildtype* for each target gene. Specifically, the relative expression levels at each time point were calculated using the equation, where ΔΔC^T^ = (C_T,Target_ − C_*Hprt*_)_Time x_ − (C_T,Target_ − C_*Hprt*_)_Time P10(*wildtype*)_.This time point was chosen as one representing a point where development is complete and before degeneration begins in the fast-degenerating models, like *Aipl1*^*-/-*^ and *Pde6b*^*rd1/rd1*^. Mean, standard error of the mean (SEM) and statistics were calculated from the 2^-ΔΔCT^. All means are stated ± SEM.

Data were analysed based on N > 3 individual retinae taken from different litters. Specifically, this included N = 4 for *wildtype* P14, all time-points in *Rho*^*-/-*^ for *Ncan* and *Ptprz1*, and *Aipl1*^*-/-*^ P10 for *Ptprz1;* N = 5 for *wildtype* P10 and 3 wks for *Vcan*; N = 7 for *Aipl1*^*-/-*^ P10 and P14 for *Vcan*; N = 8 for *Aipl1*^*-/-*^ 3 weeks for *Vcan*; and N = 3 for all other measurements. Statistical tests were performed using Graph Pad Prism v9 software. Two-way ANOVA was used to assess the differences across time within models (*red asterisk* in the graphs), one-way ANOVA with Bonferroni’s multiple comparison test for inter-group comparisons (*black asterisk*), and parametric t-tests (unpaired) for comparisons against age-matched *wildtype* controls, *blue asterisk*). Statistical significance is presented in the figures as follows: *p < 0.05, **p < 0.01, ***p < 0.001, ****p < 0.0001, ns; non-significant.

## Results

Changes in expression of four CSPGs commonly expressed in the CNS, Neurocan, Aggrecan, Phosphacan and Versican, were assessed across three different murine models of inherited blindness and in age-matched *wildtype* mice. In order to qualitatively assess the regional changes protein distribution in each model we used immunohistochemistry (IHC) and examined both the superior and inferior retina and equatorial regions, adjacent to the optic nerve (see schematics in each figure) in early, mid and advanced stages of degeneration. For quantitative analysis, real-time (RT-)qPCR was used to examine the levels of mRNA expression in whole neural retina, without RPE, over time. We first describe the patterns of expression of all four CSPGs in the normal *wildtype* mouse across time, from P10 through to 6 months of age, and then how these expression patterns are altered by retinal degeneration.

### Aggrecan, Versican, Neurocan and Phosphacan show distinct changes in expression in later stages of maturation and across adulthood in wildtype retinae

RT-qPCR revealed striking differences in the pattern of expression of *Acan*, *Vcan, Ncan* and *Ptprz1* mRNA across the later stages of postnatal development and adulthood. These trends are summarised in Fig. 1e and Table 2. Specifically, *Acan* showed a biphasic pattern of expression in *wildtype* mice (Fig. 1a); expression increased significantly between P10 and 3 weeks of age (fold change, 2.35 ± 0.49; Table 2), which was followed by a significant decrease between 3 and 6 weeks. Expression levels remained consistently low thereafter, up to 6 months of age (latest timepoint examined). While comparisons of expression levels by IHC should be assessed cautiously, a similar trend was seen in the IHC, with immunolabelling being low at P10, increasing substantially between P10 and P14 and remaining fairly constant throughout adulthood. With respect to its distribution within the retina, Aggrecan immunolabelling was particularly evident in the outer plexiform layers (OPL), inner plexiform layer (IPL) and ganglion cell layer (GCL), with relatively little staining in the outer nuclear layers (ONL) (Fig. 1g; Supplementary Figs. S1, S2). Note that for clarity of presentation, main figures show representative images of the central, superior retina (Fig. 1f); equivalent panels for the central, inferior retina for all CSPG immunostainings are shown in Supplementary Information (Supplementary Figs. Fig. S1–S4, S6, S7).

**Table 2:** Relative values of *Acan*, *Vcan*, *Ncan* and *Ptprz1* mRNA expression across time and models.

Model/time point	*Acan*	*Vcan*	*Ncan*	*Ptprz1*
*wildtype*/P10	1.01 ± 0.13	1.21 ± 0.45	1.02 ± 0.15	1.02 ± 0.14
*wildtype*/P14	1.44 ± 0.06	0.56 ± 0.24	0.36 ± 0.10	0.41 ± 0.02
*wildtype*/3 weeks	2.35 ± 0.49	0.81 ± 0.37	0.15 ± 0.04	0.17 ± 0.03
*wildtype*/6 weeks	0.99 ± 0.16	1.23 ± 0.03	0.01 ± 0.01	0.07 ± 0.02
*wildtype*/3 months	1.02 ± 0.05	0.08 ± 0.00	0.03 ± 0.01	0.04 ± 0.00
*wildtype*/6 months	0.85 ± 0.07	0.09 ± 0.01	0.02 ± 0.01	0.03 ± 0.01
*Aipl1*^*-/-*^/P10	0.87 ± 0.06	0.72 ± 0.37	0.99 ± 0.23	0.72 ± 0.12
*Aipl1*^*-/-*^/P14	2.27 ± 0.35	1.68 ± 0.59	0.36 ± 0.16	0.66 ± 0.12
*Aipl1*^*-/-*^/3 weeks	3.67 ± 0.45	1.47 ± 0.68	0.16 ± 0.05	0.28 ± 0.04
*Pde6b*^*rd1/rd1*^/P10	0.62 ± 0.13	0.53 ± 0.08	0.47 ± 0.14	0.58 ± 0.02
*Pde6b*^*rd1/rd1*^/3 weeks	2.81 ± 0.37	1.32 ± 0.64	0.02 ± 0.00	0.15 ± 0.04
*Pde6b*^*rd1/rd1*^/6–7 weeks	5.34 ± 0.08	0.23 ± 0.07	0.07 ± 0.04	0.05 ± 0.00
*Rho*^*-/-*^/P10	1.75 ± 0.30	0.60 ± 0.01	0.23 ± 0.13	0.55 ± 0.10
*Rho*^*-/-*^/3–4 weeks	2.29 ± 0.25	0.49 ± 0.09	0.08 ± 0.01	0.07 ± 0.01
*Rho*^*-/-*^/6 weeks	1.71 ± 0.04	0.27 ± 0.01	0.09 ± 0.02	0.07 ± 0.01
*Rho*^*-/-*^/3 months	1.58 ± 0.04	0.37 ± 0.02	0.10 ± 0.02	0.08 ± 0.01

Data shown is fold change ± SEM, relative to *wildtype* at P10.

Levels of *Vcan* mRNA showed greater inter-sample variability than that of the other CSPGs. However, expression was broadly similar between P10 and 6 weeks of age, and lower at 3 and 6 months of age, although this was not a statistically significant reduction (Figs. 1b, e, g; Table 2), due to inter-sample variations. IHC showed that Versican is present in all the layers of the retina, with particularly strong labelling in the photoreceptor inner/outer segment region (IS/OS) region and the OPL (Fig. 1g; Supplementary Figs. S1, S2). Staining intensity did not change markedly over time in *wildtype* mice, either across the later stages of maturation or during adulthood, although did appear weaker at 3 and 6 months (Fig. 1g, Supplementary Figs. S1, S2), consistent with the PCR data.

*Ncan* mRNA was already present at P10, but expression reduced dramatically within the first three weeks of life in *wildtype* mice, with a relative value (compared to P10 *wildtype*) of 0.15 (± 0.04) by 3 weeks and 0.01 (± 0.01) at 6 weeks of age, a level maintained up until at least 6 months of age (Fig. 1c, e; Table 2). Neurocan can undergo proteolytic cleavage of the intact molecule, resulting in smaller isoforms; the C-terminal fraction, known as Neurocan-C, and two variants of the N-terminal end, termed Neurocan-N. We therefore used two antibodies, one that detects Neurocan-C and another that detects both forms of Neurocan-N (Fig. 1g, Supplementary Figs. S1, S2). In the P10 *wildtype* retina, both Neurocan-C and Neurocan-N were expressed throughout all layers of the retina (Fig. 1g, Supplementary Figs. S1, S2). Both forms were particularly highly expressed in the OPL and at the apical side of the retina, particularly in the region of the photoreceptor inner segments, and at the basal side of the retina, in the GCL. This distribution pattern and level of expression was maintained until 3 weeks, but staining decreased dramatically between 3 and 6 weeks and remained low thereafter (Fig. 1g, Supplementary Figs. S1, S2).

Next, we investigated the expression levels and distribution of Phosphacan. Phosphacan is a splice variant of the receptor protein tyrosine phosphatase (also known as PTP-zeta, *Ptprz1*). Consistent with other reports (Inatani et al*.* 2000; Leung et al*.* 2004), we found that *Ptprz1* expression was highest at P10 and declined thereafter, with a relative value of 0.17 (± 0.03) by 3 weeks and 0.03 (± 0.01) at 6 months of age (Fig. 1d, e, Table 2). IHC showed that Phosphacan immunolabelling was most evident in the GCL, at the apical margin of the OPL and in the photoreceptor inner segment region, with weak staining in the INL and ONL (Fig. 1g). Similar to Neurocan, immunolabelling for Phosphacan decreased markedly after the first 3 weeks, with very little staining evident from 6 weeks of age in *wildtype* retinae (Fig. 1g, Supplementary Figs. S1, S2).

Together, these data show that the four CSPG core proteins show very different and distinct patterns of expression both with respect to their distribution in the retina and the amount of each core protein expressed over time in the normal wildtype adult retina. We next examined how expression of these four core proteins changes in three different models of inherited retinal degeneration. Each model was examined at early, mid and advanced stages of degeneration and compared against the relevant age-matched wildtype controls.

### Aggrecan expression is markedly increased in degeneration in Pde6b^rd1/rd1^ mice

Among the CSPGs we examined, Aggrecan expression showed the most distinct changes in expression both compared to *wildtype* and between models. In *Aipl1*^*-/-*^ and *Pde6b*^*rd1/rd1*^ mice, *Acan* mRNA increased between P10 and 3 weeks, following a similar pattern to wildtype. Notably, at middle stages of degeneration in *Aipl1*^*-/-*^ and advanced stage in *Pde6b*^*rd1/rd1*^ mice, *Acan* expression was significantly higher when compared to age-matched *wildtype* (Fig. 2d, e). The increase in *Acan* expression was most evident in *Pde6b*^*rd1/rd1*^ mice, with a relative value of 5.34 (± 0.08) at advanced stage (6 weeks) compared to 0.87 (± 0.06) at early stage (P10) degeneration in the same model (Fig. 2e, Table 2). This also yielded a highly significant increase in expression compared to age-matched (6 week) *wildtype* controls (5.34 ± 0.08 and 0.99 ± 0.16, respectively; Fig. 2e, Table 2). The overall distribution patterns of Aggrecan immunolabelling in *Aipl1*^*-/-*^ and *Pde6b*^*rd1/rd1*^ were similar to that of *wildtype,* being most evident in the OPL, inner nuclear layer (INL), IPL and GCL, although increases in labelling was most noticeable in the IPL (Fig. 2a, b, Supplementary Fig. S3).

Surprisingly, *Rho*^*-/-*^ mice, which exhibit a slower rate of degeneration compared to either *Aipl1*^*-/-*^ or *Pde6b*^*rd1/rd1*^, presented a different pattern of Aggrecan expression. *Acan* mRNA levels followed the same pattern as seen in development, reducing between 4 and 6 weeks of age, and lower at 6 months of age (Figs. 1b, 2f, Supplementary Fig. S8a). However, similar to *Aipl1*^*-/-*^ and *Pde6b*^*rd1/rd1*^ mice*,* expression was elevated at mid and advanced stages of degeneration, compared to age-matched *wildtype* mice (Figs. 1b, 2f). IHC revealed a pattern of immunolabelling very similar to that of *wildtype* and no degeneration-associated changes in the localisation of expression were observed (Fig. 2c, Supplementary Fig. S3).

### Versican expression in degeneration is similar to the pattern of expression in wildtype retina across time but demarcates Müller glial processes in Rho^-/-^ mice

In the disease models, *Vcan* mRNA expression remained largely unchanged across the respective time points examined for each model (Fig. 3d–f, Supplementary Fig. S8b). There were weak trends for an increase in *Vcan* expression between P10 and 3 weeks in *Aipl1*^*-/-*^ (Fig. 3d) and a peak in expression at 3 weeks (compared to P10 and 6 weeks) in *Pde6b*^*rd1/rd1*^ (Fig. 3e), reflecting the relevant IHC data, but these were not statistically significant due to large inter-sample variation. In *Rho*^*-/-*^ retinae, *Vcan* mRNA expression showed a trend for reducing expression with time (Fig. 3f). Notably, *Vcan* expression was significantly higher in more advanced stages of degeneration in *Rho*^*-/-*^, but was similar to, or even lower than, age-matched *wildtype* between P10 and 6 weeks. Versican maintained a similar distribution as seen in *wildtype* across time, even in the later stages of degeneration in both *Aipl1*^*-/-*^ and *Pde6b*^*rd1/rd1*^*,* with expression predominantly located in the two plexiform layers, INL and GCL (Fig. 3a, b, Supplementary Fig. S4). Conversely, in *Rho*^*-/-*^ the immunolabelling for Versican was stronger in 3 months old *Rho*^*-/-*^ retinae, compared both to earlier time points and to age-matched *wildtype* controls; labelling was most evident in the ONL and IS/OS at this stage (Fig. 3c, Supplementary Fig. S4). Versican was also more noticeable in the INL and IPL at middle time-points in *Pde6b*^*rd1/rd1*^ mice and at later time-points in *Rho*^*-/-*^ mice, something not seen in age-matched *wildtype* mice (Fig. 3b, c, Supplementary Fig. S4).

As noted above, main figures show representative images of the central, superior retina only. However, we also examined the entire retina in all cases for other spatially restricted changes. In the main, there were no obvious region-specific differences, but we did observe a disease- and region-specific change in Versican expression. Versican was found associated with Müller glial-like processes in the peripheral retina of *Rho*^*-/-*^ mice from 6 weeks onwards (Fig. 4a). Co-staining with Müller specific markers confirmed the identity of the Versican-positive processes (Fig. 4b). This observation was specific to Versican and *Rho*^*-/-*^ and was not observed for any other model or CPSG we examined (see Supplementary Figs. S1–S7). Given this apparently glia-specific pattern of Versican expression, we also examined whether Versican was expressed by Iba1-posiitive microglia cells (Supplementary Fig. S5). Co-staining was not possible due to incompatible labelling protocols; however, comparison of the patterns of Iba1 and Versican immuno-labelling does not indicate colocalization, at least within the ONL, OPL, INL and IPL. However, since some microglia somata may be located in the GCL and nerve fiber layer, we cannot rule out the possibility that these may express Versican in the later stages of degeneration in the periphery of *Rho*^*-/-*^ mice.

### Neurocan expression in degeneration is similar to the pattern of expression in wildtype retina across time

Perhaps surprisingly in view of the reported upregulation of Neurocan in other regions of the brain and the optic nerve following injury^14,18,19,30^, qRT-PCR analysis revealed no indication of an increase in *Ncan* mRNA levels in any of the three models of progressive degeneration, compared to age-matched *wildtype* retinae. Both *Aipl1*^*-/-*^ and *Pde6b*^*rd1/rd1*^ mice showed the same profile of expression across age, with highest levels seen at P10 and decreasing with age, in line with *wildtype* mice (Fig. 5d, e; Table 2). However, the relative levels of *Ncan* at P10 were lower than that of age-matched *wildtype* mice in both *Pde6b*^*rd1/rd1*^ and *Rho*^*-/-*^ mice and expression dropped further after P10 (Fig. 5e, f, Supplementary Fig. S8c). This initial reduction was most apparent in *Rho*^*-/-*^ (Fig. 5f) although *Ncan* mRNA levels then remained stable between 3–4 weeks and 3 months of age in *Rho*^*-/-*^ mice. This differs from *wildtype* mice where the levels continued to drop after 6 weeks of age, yielding a significant difference between *Rho*^*-/-*^ and age-matched *wildtype* mice (Fig. 5f).

The spatial distribution of both Neurocan-C and Neurocan-N is similar in the different models of degeneration as in the *wildtype* eye, with expression predominantly locating to the OPL and outer margin of the retina in the segment region (Fig. 5a, b, c, Supplementary Figs. S1, S6). Neurocan-N levels were broadly similar in all disease models, although immunolabelling was consistently stronger in the *Aipl1*^*-/-*^ mice than that observed in age-matched *wildtype* mice (Fig. 5a, Supplementary Figs. S6). Conversely, immunolabelling for Neurocan-C was typically weaker in mid to advanced stages of degeneration in both *Aipl1*^*-/-*^ and *Pde6b*^*rd1/rd1*^ eyes, compared to *wildtype* (Fig. 5a, b, Supplementary Figs. S1, S6). In *Rho*^*-/-*^, Neurocan-N remained fairly consistent between 3–4 weeks and 3 months of age (earlier time points not examined), while immunostaining intensity increased over the same period for Neurocan-C (Fig. 5c, Supplementary Fig. S6). Both Neurocan-C and Neurocan-N were distributed in a pattern similar to that seen in the *wildtype* retina, throughout all the layers of retina, with increased deposition of Neurocan-C at mid to advanced stages in GCL, IPL, INL and OPL (Fig. 5c, Supplementary Fig. S6). No consistent differences between the superior or inferior retina in the patterns of Neurocan (C or N) staining were observed in any of the models (Figs. 5a, b, c, Supplementary Figs. S1, S6).

### Phosphacan expression in degeneration is similar to the pattern of expression in wildtype retina across time

In all disease models, *Ptprz1* mRNA levels followed the same expression profile as *wildtype* (Fig. 1d), with expression of *Ptprz1* decreasing significantly over time (Fig. 6d, e, f). Of note, and similar to *Ncan*, mRNA levels of *Ptprz1* in *Pde6b*^*rd1/rd1*^ at P10 was statistically lower than that of the same age in *wildtype* mice (Fig. 6e). The same tendency was also observed in *Aipl1*^*-/-*^ and *Rho*^*-/-*^ mice but was not statistically significant (Fig. 6d, f, Supplementary Fig. S8d). In adult *Rho*^*-/-*^ mice, *Ptprz1* expression remained low and unchanged over time and degeneration, like age-matched adult *wildtype* retina (Fig. 6f). *Aipl1*^*-/-*^ and *Pde6b*^*rd1/rd1*^ mice showed a similar distribution of Phosphacan protein as that seen in *wildtype* mice, although labelling was more intense in the OPL and RPE, and comparatively low in the GCL (Fig. 6a, b, Supplementary Fig. S7). Of note, *Rho*^*-/-*^ mice showed little or no change in immunolabelling for Phosphacan and the distribution remained similar to age-matched *wildtype* retinae throughout (Fig. 6c, Supplementary Fig. S7).

## Discussion

CSPGs are widely reported to be upregulated following neuronal injury. This study demonstrates that this is an oversimplification and that there is significant heterogeneity in response to progressive neuronal degeneration, with different CSPG core proteins exhibiting very distinct patterns of expression, even between models with similar rates of neurodegeneration. Previously, we examined global changes in CSPG protein deposition using the CS-56 antibody^24^ and found that deposition broadly increased over time in *wildtype* and *Rho*^*-/-*^ and decreased in *Pde6b*^*rd1/rd1*^. However, such an approach may mask different changes in individual CSPGs. Our present data examining mRNA levels for four CSPG core proteins showed decreasing expression of *Ncan* and *Ptprz1* in all models, contrasted by a significant increase of *Acan* in *Pde6b*^*rd1/rd1*^ and *Aipl1*^*-/-*^ retinae, and no significant change of *Vcan* in any model, including *wildtype*. These differences highlight the importance of studying specific CSPGs, as well as global changes. This study is the first comprehensive assessment of specific CSPGs in murine models of progressive retinal degeneration. It also provides the first comparative assessment of multiple CSPGs across time in the late postnatal and adult wildtype retina.

Many factors affect the relative levels of a given CSPG core protein, including synthesis and degradation. CSPG-degrading enzymes such as MMP (matrix metalloproteinases) and ADAMTS (A Disintegrin and Metalloproteinase with Thrombospondin Motifs)^31–33^ degrade different CSPGs with different efficiencies. The expression of these enzymes can change significantly, both during development and in ocular diseases, such as diabetic retinopathy, myopic chorioretinal atrophy, cataract and retinitis pigmentosa^34–38^, and alongside Müller glia proliferation^39^. Other CSPGs that were not assessed in the current study may also contribute to the global changes in CSPG deposition, as assessed with CS-56.

Understanding how CSPGs exert their actions on neurite outgrowth is essential if we are to manipulate the diseased microenvironment in order to make it more permissive for regeneration. In our present work, Aggrecan and Versican were most prevalent, particularly in degeneration, and with aging, while Neurocan and Phosphacan were much less evident. Aggrecan and Versican each contain more GAG chains (~ 100 and 5–23, respectively) than either Neurocan or Phosphacan (< 5)^40^, suggesting that these CSPGs may present an inhibitory chemical barrier to synaptic plasticity during degeneration and/or regeneration. Enzymatic digestion of GAG chains can promote axonal regeneration and plasticity following CNS damage^7^. Consistent with this, treatment of recipient retinas with Chondroitinase ABC improved donor-host interactions following cell transplantation^1,41–44^, while viral diffusion and transduction efficiency were improved by co-application of ChABC with viral vector delivery^45–47^. Enzymatic degradation is not without its limitations, however^48,49^ and significant inhibitory function is retained by the core protein, even after GAG digestion^9^.

Of the CSPGs examined, Aggrecan demonstrated the greatest heterogeneity across disease models. In the normal adult eye, it is thought to play a role in the maintenance of the laminar structure of the retina^50–52^. In mice, refinement of synaptic sub-laminae is not complete until P21^53^. Aggrecan expression is seen from embryonic stages and increases with age; it is widespread, even in the normal adult retina, particularly in the synaptic layers^21,22^. Consistent with this, we found *Acan* expression to increase significantly between P10 and 3 weeks of age and remain at a similar level thereafter in the *wildtype* retina; immunolablling for Aggrecan increased markedly between P10 and P14 and was most evident in the inner and outer plexiform layers. Together, these changes indicate a role for Aggrecan in the establishment and maintainance of laminar structure. Previous reports examining the developing rat and mouse retina have reported the expression of both Neurocan and Phosphacan from E13-17, peaking around P7^20,21,54,55^. Given that the primary focus of this study was to examine expression across progressive degeneration, we did not look earlier than P10. However, consistent with these reports, we found that the expression of Neurocan and Phosphacan was highest in all models at P10 and declined thereafter.

Aggrecan’s upregulation following acute injuries, such as spinal cord crush^13,37^, is well reported. It impedes the regeneration of both endogenous and transplanted neurons^12,13^. Its expression in progressive degeneration is less well characterised but it is upregulated in rats with retinal dystrophy^52^ and induced retinal ischemia^26^. Similarly, we found that Aggrecan expression was dramatically upregulated in the *Aipl1*^*-/-*^ and *Pde6b*^*rd1/rd1*^ models of rapid photoreceptor degeneration, but, surprisingly, not in the more slowly degenerating *Rho*^*-/-*^, which undergoes a slower rate of degeneration. These differences are intriguing and further exploration of Aggrecan in other models of slow-to-moderate degeneration would be of interest to establish whether rate of degeneration is a determining factor. Some ADAMTS genes, including the aggrecanase ADAMTS5 (also known as ADAMTS11), are reported to be upregulated in AMD and are thought to influence retinal pathology by proteolytic modification of the retinal extracellular matrix^56^. Of note, studies in the barrel cortex have shown that of PNNs are lost during sensory deprivation and that this is mediated by a reduction in the expression of Aggrecan^57,58^. Hence, targeted reduction of Aggrecan may be beneficial for therapeutic strategies that require the formation of new synapses.

Although Versican expression was broadly similar in *wildtype, Aipl1*^*-/-*^ and *Pde6b*^*rd1/rd1*^ retinae, we found notable region-specific changes in expression in the *Rho*^*-/-*^ mouse. In our study, Versican + /Gfap + Müller glial processes were observed, but only in the peripheral retina of *Rho*^*-/-*^ mice and at later stages of degeneration. Iba1 + microglia in the same region did not appear to co-label with Versican. The differential distribution of Versican, both with respect to a sub-population of Müller glia within a given retina and also between models is worthy of further investigation. Note that the rod photoreceptors in the *Rho*^*-/-*^ model are non-functional from their genesis onwards so it is conceivable that some of the model-specific changes observed in this study may reflect a developmental alteration in response to the absence of rod function. Cone function is, however, normal in these mice. In order to assess all isoforms of Versican, we used an antibody detecting the G3 domain to assess regional changes and chose PCR primers corresponding to the same sequence used to raise the antibody (exon 14–15). The G3 domain has been reported to play a key role in the secretion of Versican from the cell^59^ and binds to several molecules including the EGF receptor, tenascin, fibronectin and integrin β1^60,61^. The G3 domain has been shown to promote neurite outgrowth and cell attachment in hippocampal neurons via an EGF receptor-mediated pathway^62^, potentially indicating a localised pro-regenerative impact on the environment in the periphery. The specific association of G3 domain with peripheral Müller glial processes, which in lower vertebrates retain a more immature, progenitor/stem cell-like phenotype, indicates local microenvironmental differences within the degenerating retina.

Neurocan is upregulated in many experimental models of acute injury in the brain^14,18,19^, optic nerve crush^30^ and in the ischaemic rat retina^63^. In contrast, we did not observe any upregulation of Neurocan with progressive retinal degeneration. Indeed, mRNA levels of *Ncan* and *Ptprz1* at P10 were actually significantly lower in *Pde6b*^*rd1/rd1*^ and *Rho*^*-/-*^, compared to age-matched *wildtype* mice. There was an accompanying reduction in Neurocan-C staining at the OPL/ONL interface as degeneration progresses in *Aipl1*^*-/-*^ and *Pde6b*^*rd1/rd1*^, while labelling for the Neurocan-N fraction remained fairly constant in all disease models. Neurocan, and its cleavage, demonstrates dynamic changes during development, injury and remodelling in the CNS; full length Neurocan was expressed in juvenile rat brain^65^ and following acute brain injury^14,65^. In contrast, only cleaved Neurocan was present in rat adult brain, and more interestingly, only N- and not C-terminal fragments were observed as a component of PNNs in adult rat cerebellum^64^. A potential role for cleaved Neurocan was indicated in a recent study showing Neurocan-N fractions are essential in Semaphorin 3F-induced dendric spine remodelling^66^. In our data, the labelling of both C- and N-terminal fractions were observed in early stages, indicating that full length fractions are likely present in the juvenile retina. Conversely, neither of the fractions were seen in significant amounts from 3 weeks of age in *wildtype* mice. Both changes are similar to those described for the brain^64^. In contrast, Neurocan-N and -C fractions predominated in *Aipl1*^*-/-*^ and *Pde6b*^*rd1/rd1*^, respectively. Similarly, Phosphacan labelling was seen at the outer margin of the OPL between P10 and 3 weeks in *wildtype*, but not in *Aipl1*^*-/-*^ and *Pde6b*^*rd1/rd1*^, retinae. Observationally, the separation of photoreceptors in the ONL and the OPL was also less well defined in both *Aipl1*^*-/-*^ and *Pde6b*^*rd1/rd1*^ mice, compared to *wildtype*. While the reduced levels may simply reflect a reduction in the number of photoreceptor neurons that may produce these proteins, the different patterns of expression seen in *wildtype* and diseased retina, together with previous studies examining Neurocan cleavage, suggest that Neurocan and Phosphacan may play a role in the establishment and maintenance of retinal lamination.

In summary, our study provides the first comprehensive characterisation of individual CSPGs and how their expression changes both with age and with progressive retinal degeneration. It also highlights an important heterogeneity in the retinal environments that arise in degeneration due to different genetic causes and the importance for researchers to characterize the specific models they use. Understanding the distinct changes in expression and distribution of individual CSPGs may help us to modulate specific diseased microenvironments, affecting synaptic plasticity, regeneration and repair.

## Supplementary Information

Supplementary Information.

## Data Availability

According to UK research council's Common Principles on Data Policy, data supporting this study will be openly available at https://github.com/RPearsonLab/CSPG-expression-in-the-retina.

## Abbreviations

CNS: Central nervous system
CSPG: Chondroitin sulphate proteoglycan
GAG: Glycosaminoglycan
GCL: Ganglion cell layer
GFAP: Glial fibrillary acidic protein
INL: Inner nuclear layer
IPL: Inner plexiform layer
IS: Inner segment
ONL: Outer nuclear layer
OPL: Outer plexiform layer
OS: Outer segment
