# Supplementary material for: A comprehensive atlas of Aggrecan, Versican, Neurocan and Phosphacan expression across time in wildtype retina and in retinal degeneration
Source: Sci Rep. 2022 May 4;12:7282. doi: 10.1038/s41598-022-11204-w (PMC9068689; doi:10.1038/s41598-022-11204-w)
Supplement: Supplementary file 1 — Supplementary Information. [file 41598_2022_11204_MOESM1_ESM.pdf]

Supplementary Tables

Supplementary Table S1. Antibodies and summary of immunohistochemistry protocols used to assess CSPGs expression. Abbreviations: NGS; Normal goat serum (Abd Serotec, Oxford UK). BSA; Bovine Serum Albumin (Sigma Aldrich, Dorset UK).

**Antibody**	**Fixation**	**ChABC treatment***	**Blocking Solution**	**Primary Antibody concentration**	**Secondary Antibody concentration**
**Neurocan, N-terminal** (Kind gift from Fawcett Group)	Unfixed	No treatment	5% NGS, 1% BSA, 0.25% Triton in PBS	1:200	Alexa 546 goat anti-rabbit (A-11071) 1:400
**Neurocan, C-terminal** (Kind gift from Fawcett Group)	Unfixed	No treatment	1% NGS, 5% Milk, 0.05% Triton in PBS	1:100	Alexa 546 goat anti-rabbit (A-11071) 1:400
**Aggrecan** (Millipore, AB1031)	Unfixed	0.01U at 37ºC for 3hr	1% NGS, 5% Milk, 0.1% Triton in PBS	1:100	Alexa 546 goat anti-rabbit (A-11071) 1:400
**Versican** (Santa Cruz, H-56)	Unfixed	0.1U at 37ºC for 3hr	1% NGS, 3% BSA, 1% Triton in PBS	1:100	Alexa 546 goat anti-rabbit (A-11071) 1:400
**Phosphacan** (DSHB, 3F8)	Unfixed	0.1U at 37ºC for 3hr	1% NGS, 3% BSA, 1% Triton in PBS	1:10 (Ab conc. 31μg/ml)	Alexa 546 goat anti-mouse (A-11018) 1:400
**GFAP** (Sigma Aldrich, G3893)	Fixed and Unfixed	No treatment	1% NGS, 3% BSA, 1% Triton in PBS	1:200	Alexa 488 goat anti-mouse (A-11001) 1:400
**Iba1** (WAKO, 019-19741)	Fixed	No treatment	1% NGS, 3% BSA, 1% Triton in PBS	1:500	Alexa 488 goat anti-rabbit (A-11008) 1:400

Supplementary Figures

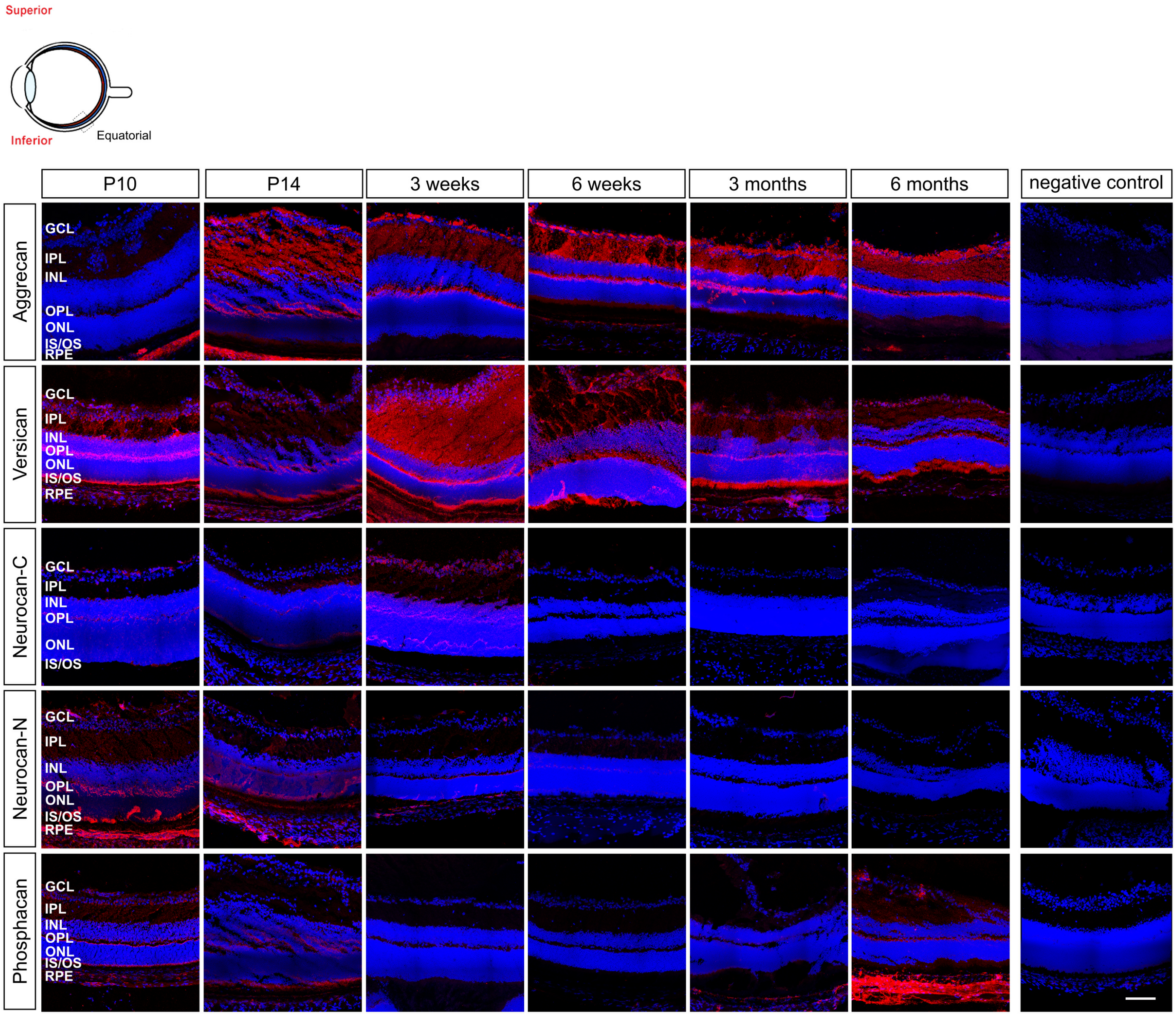

Figure S1. Aggrecan, Versican, Neurocan and Phosphacan in the inferior retina of *wildtype* mice over time. Immunolabelling for Aggrecan, Versican, Neurocan and Phosphacan *(red)* over time. When expressed, Neurocan-C and -N fractions and Versican were sparsely distributed throughout all the layers of retina. Phosphacan and Aggrecan were restricted mostly to the GCL, IPL and OPL. No discernible difference was observed when compared to the superior retina (see Fig. 2). Images are confocal maximum intensity projection (MIP) images taken in the inferior retina in the equatorial region. Scale bar, 100μm. ONL – outer nuclear layer; OPL – outer plexiform layer; INL – inner nuclear layer; IPL – inner plexiform layer; GCL – ganglion cell layer. Nuclei are counter stained with Dapi - *blue*; CSPGs -*red*.

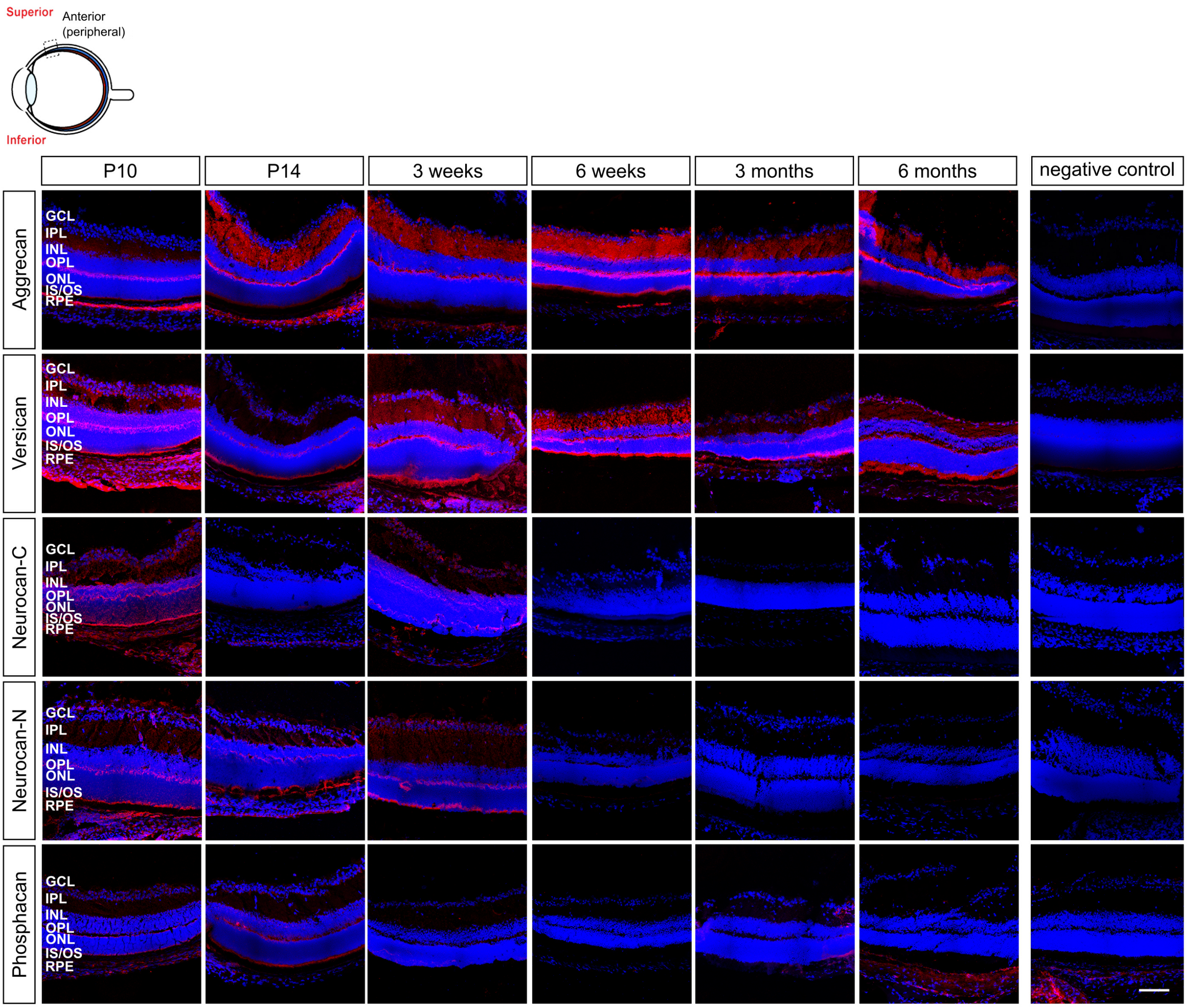

Figure S2. Aggrecan, Versican, Neurocan and Phosphacan in *wildtype* mice over time in anterior margin (peripheral retina). Immunolabelling for Aggrecan, Versican, Neurocan and Phosphacan *(red)* over time. Similar to labelling in both the superior and inferior retina in the equatorial region (see Fig 2, Fig S1), Neurocan-C and -N fractions and Versican were sparsely distributed throughout all the layers of the peripheral retina when expressed. Phosphacan and Aggrecan were restricted mostly to the GCL, IPL and OPL. Images are confocal MIPs taken in the superior anterior margin (peripheral retina). Scale bar, 100µm. ONL – outer nuclear layer; OPL – outer plexiform layer; INL – inner nuclear layer; IPL – inner plexiform layer; GCL – ganglion cell layer. Nuclei are counter stained with Dapi - *blue*; CSPGs -*red*.

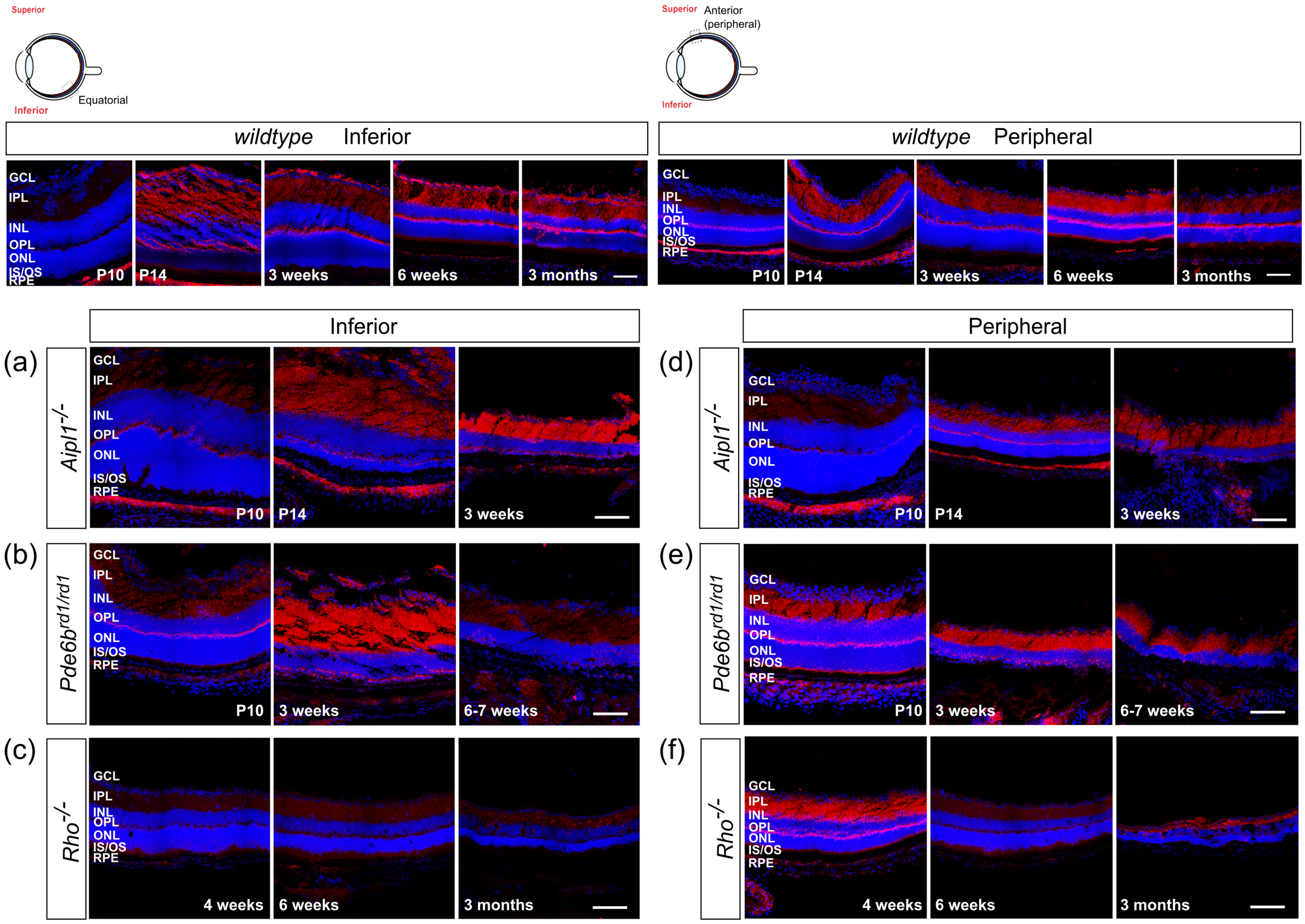

Figure S3. Aggrecan in inferior retina in the equatorial region and anterior margin (peripheral). (a)-(c) Inferior retina in the equatorial region, (d)-(f) anterior margin of the retia (peripheral). Immunolabelling for Aggrecan (*red*) was mainly seen in the OPL, IPL, and GCL, with limited staining in ONL in all models. No discernible differences were observed when compared to the superior retina in the equatorial region (see Fig. 3). Images shown are confocal MIPs taken either in the inferior retina the equatorial region or the superior retina in the anterior margin (peripheral retina). Scale bar, 100µm. ONL – outer nuclear layer; OPL – outer plexiform layer; INL – inner nuclear layer; IPL – inner plexiform layer; GCL – ganglion cell layer. Nuclei are counter stained with Dapi - *blue*; Aggrecan -*red*.

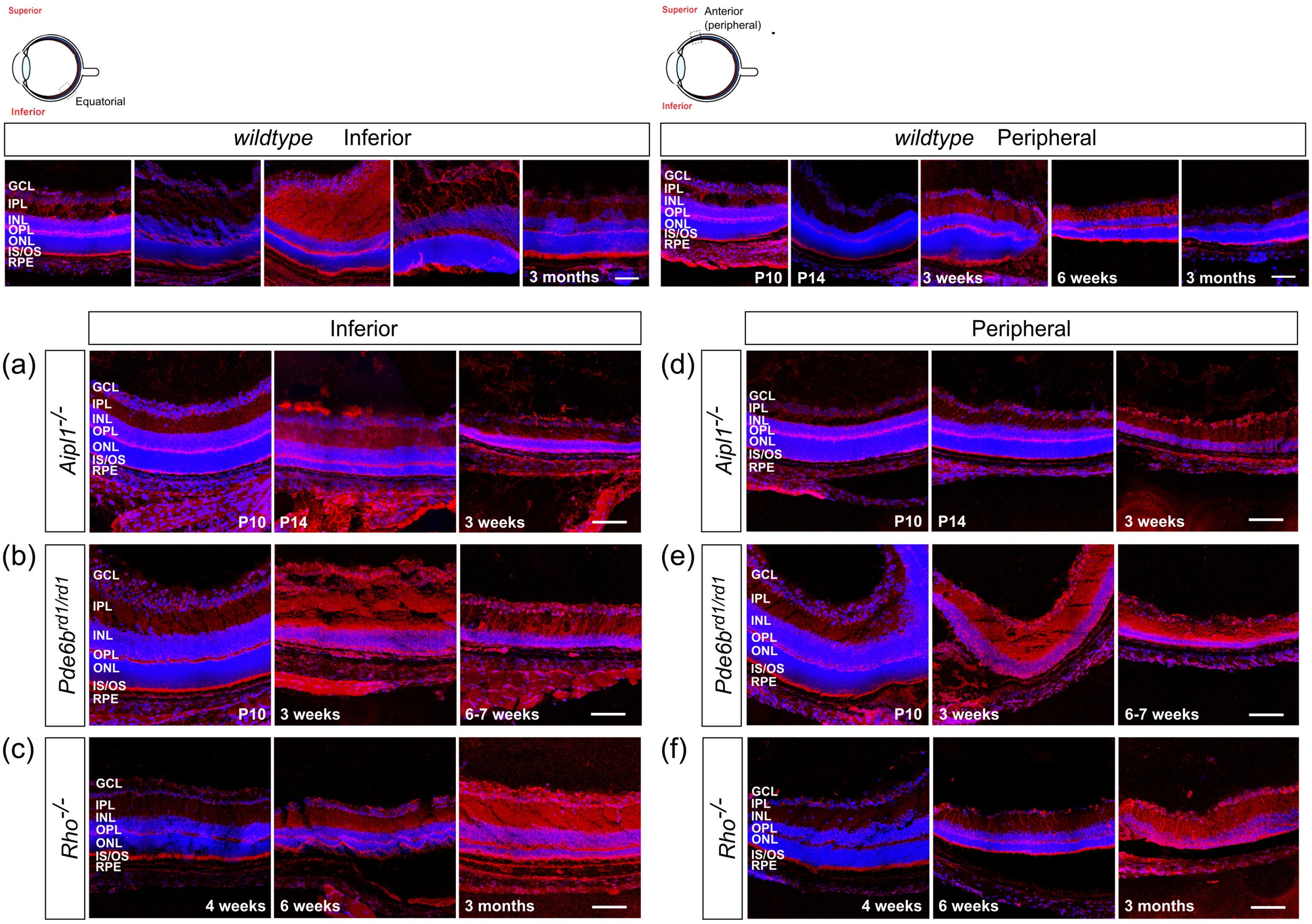

Figure S4. Versican in inferior retina in the equatorial region and anterior margin (peripheral). (a)-(c) Inferior retina in the equatorial region, (d)-(f) anterior margin of the retia (peripheral). Immunolabelling for Versican (*red*) was detected in all layers of the retina in *wildtype*, *Aipl1*$^{-/-}$ and *Pde6b*$^{rd1/rd1}$ with relatively strong signals in the INL and IPL, particularly in mid and advanced stages of *Pde6b*$^{rd1/rd1}$ (b),(e) and advanced-stage *Rho*$^{-/-}$ (c),(f) retinae. Versican^{+ve} Müller glial processes were specific to the peripheral retina of *Rho*$^{-/-}$ mice in mid to advanced stages of degeneration (f) and this pattern of labelling was not observed in the other models examined**.** Images shown are confocal MIPs taken either in the inferior retina in the equatorial region or the superior retina in anterior margin (peripheral retina). Scale bar, 100µm. ONL – outer nuclear layer; OPL – outer plexiform layer; INL – inner nuclear layer; IPL – inner plexiform layer; GCL – ganglion cell layer. Nuclei are counter stained with Dapi - *blue*; Versican -*red*.

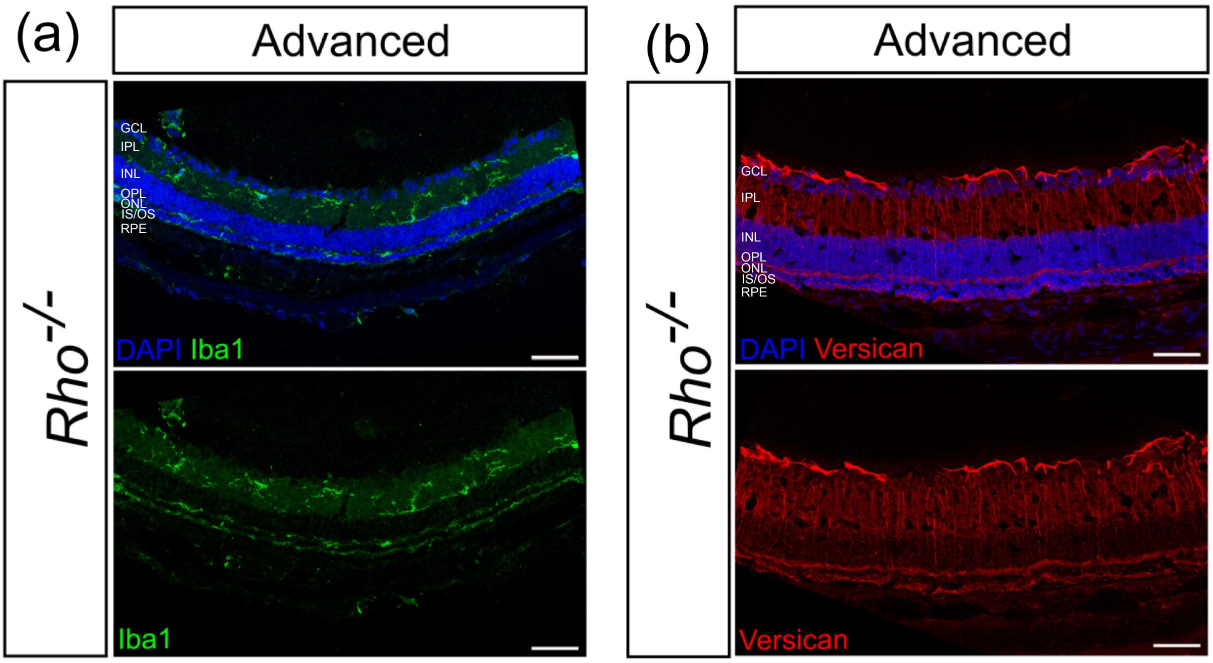

Figure S5. Versican does not colocalize with Ib1+ microglia in the peripheral retina in advanced stage degeneration of *Rho*$^{-/-}$ mice. (a) Immunolabelling of microglial marker, Iba1 (Ionized calcium binding adaptor molecule 1; *green*) exhibits the typical ramified profile of activated microglia throughout the ONL, OPL, INL and IN, but not radial processes in the peripheral retina (fixed tissue). (b) Radial Versican+ processes (*red*) in the peripheral retina (unfixed tissue) are shown for comparison. Scale bar, 100µm. Nuclei are counter stained with Dapi (*blue*).

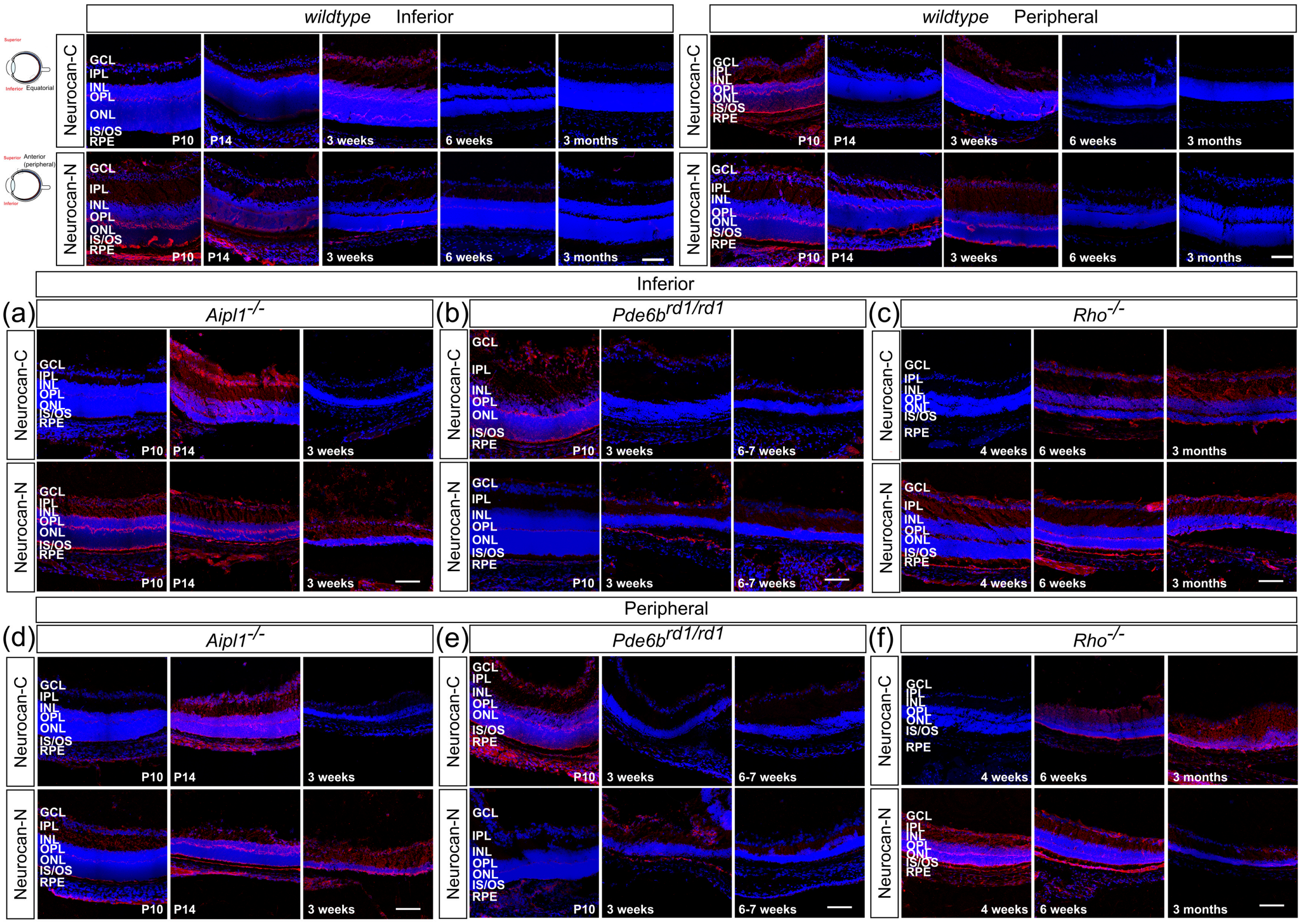

Figure S6. Neurocan in inferior retina in the equatorial region and anterior margin (peripheral). (a)-(c) Inferior retina in the equatorial region, (d)-(f) anterior margin of the retia (peripheral). Neurocan immunolabelling (*red*) was distributed throughout all the layers of the retina in all models. No marked difference was observed compared to the superior retina in the equatorial region (see Fig. 6). (a)(b)(d)(e), Similar to the superior retina (Fig. 6), immunolabelling of the Neurocan C-terminal fraction typically decreased in intensity in *Aipl1*$^{-/-}$ and *Pde6b*$^{rd1/rd1}$ mice with time, while that of N-terminal fraction remained constant in these models. (c)(f) Immunolabelling for both Neurocan-C and -N were largely unchanged across degeneration in *Rho*$^{-/-}$ mice. Images shown are confocal MIPs taken in the inferior retina in the equatorial region. Scale bar, 100µm. ONL – outer nuclear layer; OPL – outer plexiform layer; INL – inner nuclear layer; IPL – inner plexiform layer; GCL – ganglion cell layer. Nuclei are counter stained with Dapi - *blue*; Neurocan - *red*.

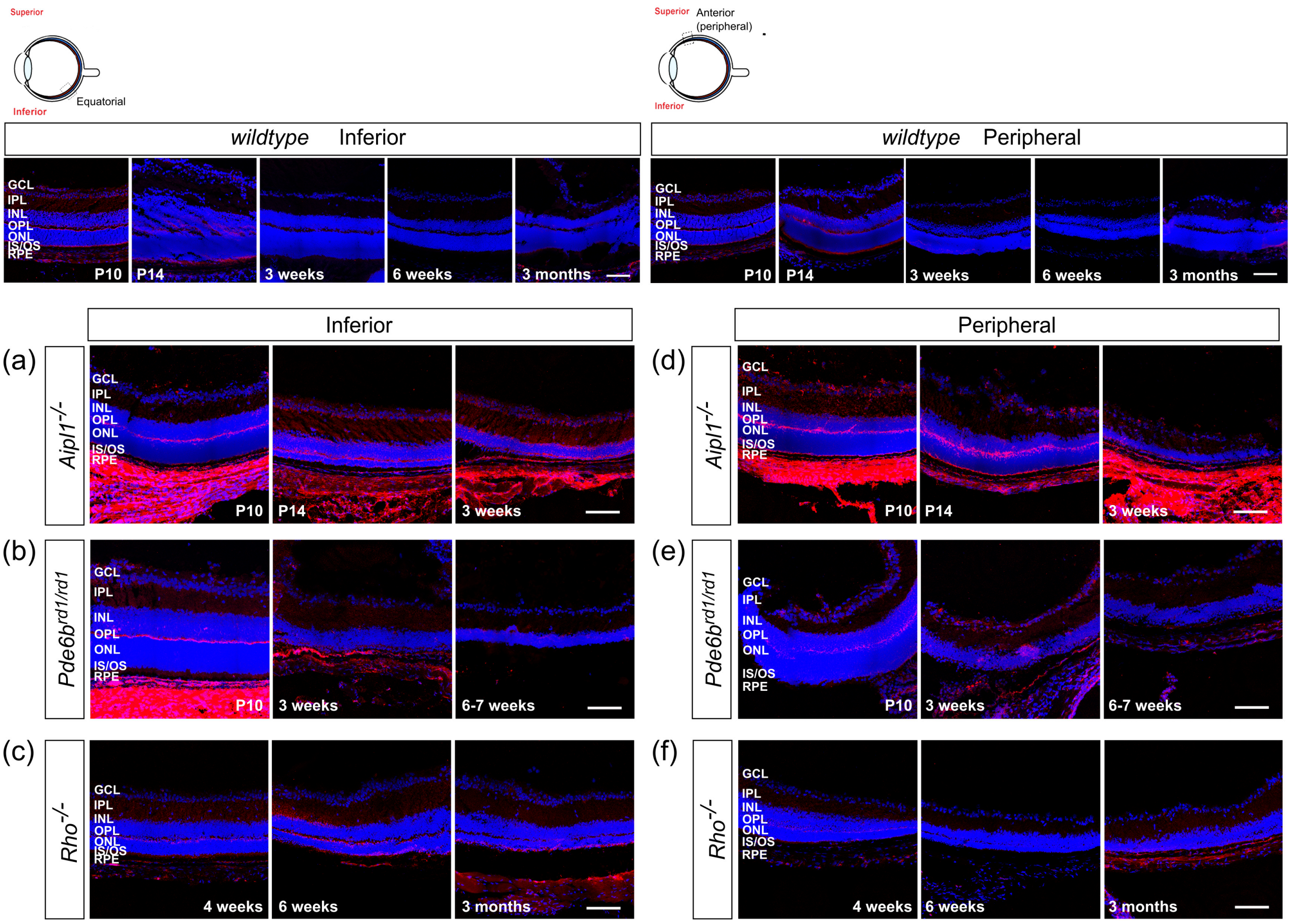

Figure S7. Phosphacan in inferior retina in the equatorial region and anterior margin (peripheral retina). (a)-(c) Inferior retina in the equatorial region. (d)-(f) anterior margin of the retia (peripheral). Immunolabelling for Phosphacan (*red*) was mostly restricted to GCL, IPL and OPL (a)(b)(d)(e) in inferior retina in P10 *wildtype*. In *Aipl1*$^{-/-}$ and *Pde6b*$^{rd1/rd1}$ mice, labelling was most prominent in the OPL and RPE, compared to age-matched *wildtype*. Unlike P10 *wildtype*, labelling at the outer margin of the OPL and the edge of the ONL itself were not noticeable in these mice. (c)(f) In *Rho*$^{-/-}$ retinae, labelling was weak at all time-points. Images shown are confocal MIPs taken in the inferior retina in the equatorial region or the superior retina in anterior margin (peripheral retina). Scale bar, 100µm. ONL – outer nuclear layer; OPL – outer plexiform layer; INL – inner nuclear layer; IPL – inner plexiform layer; GCL – ganglion cell layer. Nuclei are counter stained with Dapi - *blue*; Phosphacan -*red*.

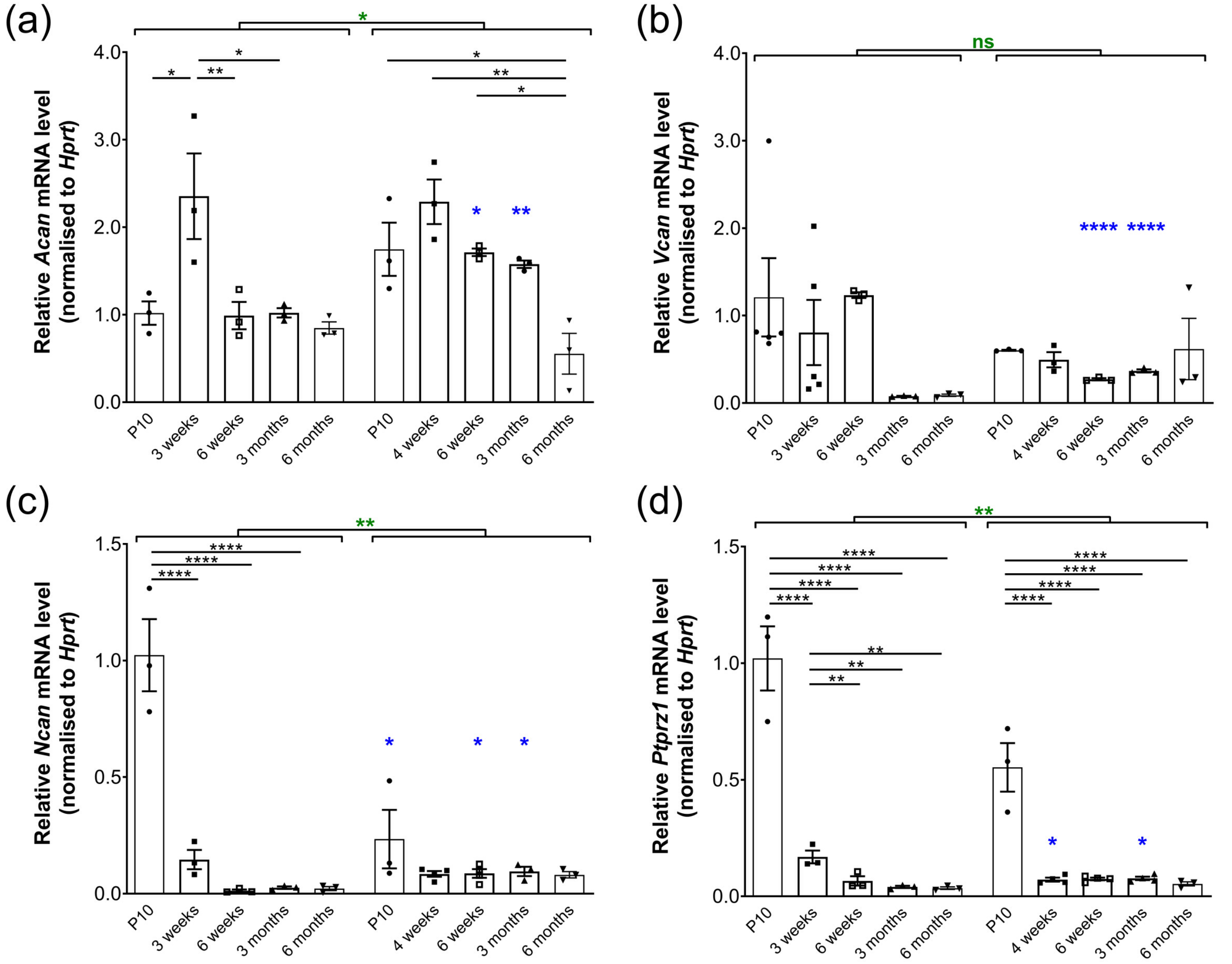

Figure S8. *Acan, Vcan, Ncan* and *Ptprz1* mRNA levels in *wildtype* and *Rho*$^{-/-}$ with early and latest time points. (a) *Acan*, (b) *Vcan*, (c) *Ncan*, and (d) *Ptprz1*. (a)(b) mRNA expression of *Acan* and *Vcan* was largely unchanged with time in *Rho*$^{-/-}$ retinae. (a) *Acan* mRNA expression in *Rho*$^{-/-}$ retinae showed a similar trend as *wildtype* mice, although small upregulation in 6 weeks and 3 months of age compared to age-matched *wildtype* mice. Unlike *Aipl1*$^{-/-}$ and *Pde6b*$^{rd1/rd1}$ retinae, *Acan* mRNA level was not increased in the latest time point, 6 months of age. (b) *Vcan* mRNA expression showed a small reduction, compared to early stages, and this reduction was the most apparent at 6 weeks old. (c) *Ncan* expression at P10 in *Rho*$^{-/-}$ retinae was markedly lower than age-matched *wildtype* mice. *Ncan* expression declined from P10 to 4 weeks of age, although this change was not statistically significant. (d) mRNA levels of *Ptprz1* in P10 in *Rho*$^{-/-}$ mice were lower in comparison to age-matched *wildtype* mice, and the expression decreased further from P10 to 4 weeks of age and remained low thereafter.
